# Hepatocyte-derived DPP4 regulates portal GLP-1 bioactivity, modulates glucose production, and when absent influences NAFLD progression

**DOI:** 10.1172/jci.insight.154314

**Published:** 2023-01-24

**Authors:** Natasha A. Trzaskalski, Branka Vulesevic, My-Anh Nguyen, Natasha Jeraj, Evgenia Fadzeyeva, Nadya M. Morrow, Cassandra A.A. Locatelli, Nicole Travis, Antonio A. Hanson, Julia R.C. Nunes, Conor O’Dwyer, Jelske N. van der Veen, Ilka Lorenzen-Schmidt, Rick Seymour, Serena M. Pulente, Andrew C. Clément, Angela M. Crawley, René L. Jacobs, Mary-Anne Doyle, Curtis L. Cooper, Kyoung-Han Kim, Morgan D. Fullerton, Erin E. Mulvihill

**Affiliations:** 1 Department of Biochemistry, Microbiology and Immunology, Faculty of Medicine, University of Ottawa, Ontario, Canada.; 2 University of Ottawa Heart Institute, Ottawa, Ontario, Canada.; 3 Centre for Infection, Immunity and Inflammation, Ottawa, Ontario, Canada.; 4 Centre for Catalysis Research and Innovation, Ottawa, Ontario, Canada.; 5 Li Ka Shing (LKS) Centre for Health Research Innovation, Department of Agricultural, Food and Nutritional Science, University of Alberta, Edmonton, Alberta, Canada.; 6 Ottawa Hospital Research Institute, Ottawa, Ontario, Canada.; 7 Department of Biology, Carleton University, Ottawa, Ontario, Canada.; 8 Division of Endocrinology & Metabolism, Department of Medicine,; 9 Division of Infectious Diseases, Department of Medicine, and; 10 Department of Cellular and Molecular Medicine, University of Ottawa, Ottawa, Ontario, Canada.; 11 Montréal Diabetes Research Group, Montréal, Québec, Canada.

**Keywords:** Inflammation, Metabolism, Fibrosis, Gluconeogenesis, Peptides

## Abstract

Elevated circulating dipeptidyl peptidase-4 (DPP4) is a biomarker for liver disease, but its involvement in gluconeogenesis and metabolic associated fatty liver disease progression remains unclear. Here, we identified that DPP4 in hepatocytes but not TEK receptor tyrosine kinase–positive endothelial cells regulates the local bioactivity of incretin hormones and gluconeogenesis. However, the complete absence of DPP4 (*Dpp4^–/–^*) in aged mice with metabolic syndrome accelerates liver fibrosis without altering dyslipidemia and steatosis. Analysis of transcripts from the livers of *Dpp4^–/–^* mice displayed enrichment for inflammasome, p53, and senescence programs compared with littermate controls. High-fat, high-cholesterol feeding decreased *Dpp4* expression in F4/80^+^ cells, with only minor changes in immune signaling. Moreover, in a lean mouse model of severe nonalcoholic fatty liver disease, phosphatidylethanolamine *N*-methyltransferase mice, we observed a 4-fold increase in circulating DPP4, in contrast with previous findings connecting DPP4 release and obesity. Last, we evaluated DPP4 levels in patients with hepatitis C infection with dysglycemia (Homeostatic Model Assessment of Insulin Resistance > 2) who underwent direct antiviral treatment (with/without ribavirin). DPP4 protein levels decreased with viral clearance; DPP4 activity levels were reduced at long-term follow-up in ribavirin-treated patients; but metabolic factors did not improve. These data suggest elevations in DPP4 during hepatitis C infection are not primarily regulated by metabolic disturbances.

## Graphical abstract

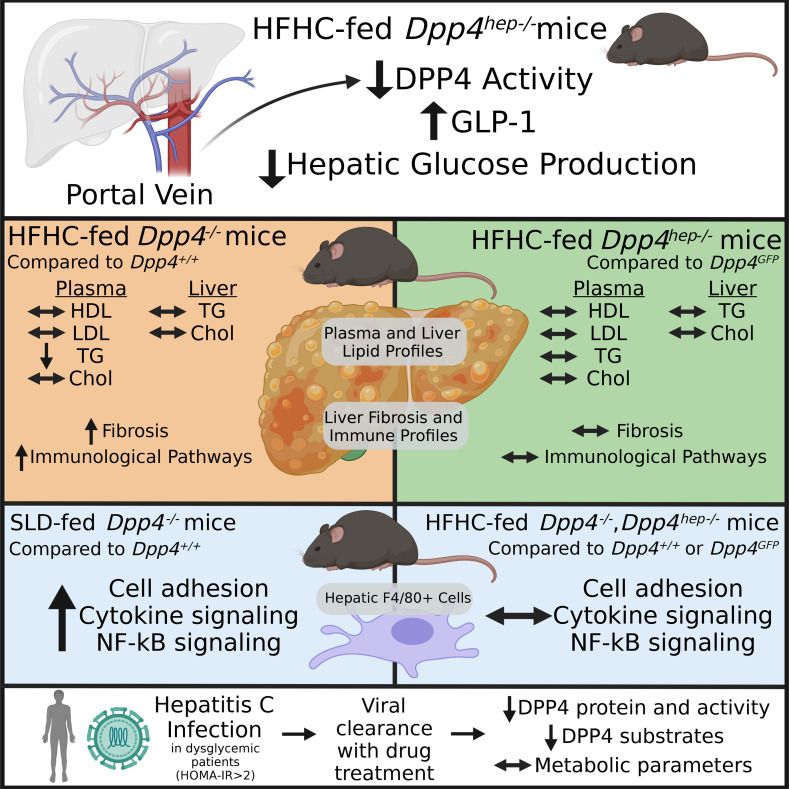

## Introduction

Type 2 diabetes (T2D) is a metabolic disease characterized by the development of hyperglycemia. Dysregulated islet hormone secretion and an inability to overcome peripheral insulin resistance are central components (1, 2). Given the increased appreciation of the reciprocal nature of dyslipidemia, obesity, dysglycemia and liver disease, including nonalcoholic fatty liver disease (NAFLD) and traditional risk factors, a new classification of metabolic (dysfunction) associated fatty liver disease (MAFLD) has emerged (3).

The secretion of incretin hormones from enteroendocrine cells in the gut epithelium, including glucagon-like peptide-1 (GLP-1) and glucose-dependent insulinotropic polypeptide (GIP), potentiates postprandial insulin secretion, a phenomenon known as the incretin effect. GLP-1 and GIP are central in coordinating nutrient intake, nutrient disposal, and satiety (4–6). In patients with T2D, there is a defect in the incretin-mediated potentiation of insulin secretion (7). Additionally, individuals with NAFLD exhibit an impaired incretin effect independent of diabetes, displaying fasting hyperglucagonemia (8) and increased hepatic gluconeogenesis (9).

The bioactivity and action of endogenous incretin hormones are limited through proteolytic cleavage and inactivation by the serine protease dipeptidyl peptidase-4 (DPP4) and renal elimination (10, 11). Enzymatically active DPP4 is present in both membrane-bound and circulating forms (12). Plasma DPP4 levels are elevated in several settings associated with metabolic dysfunction, such as obesity (13, 14); chronic liver disease, including NAFLD and hepatitis C infection (HCV) (12, 15, 16); as well as type 1 diabetes (17) and T2D (18, 19). Increased *DPP4* expression in the liver positively correlates with the degree of steatosis and NAFLD (20, 21). Studies in mice using several tissue-specific targeting strategies have confirmed that the elevation of circulating DPP4 in obesity is liver derived (22–24), suggesting that DPP4 produced in the liver may primarily contribute to the progression of NAFLD (25). Systemic inhibition of DPP4 decreases blood glucose by reducing hepatic glucose production (HGP) in patients with T2D (26). Given the success of incretin-based drugs in treating both diabetes and obesity, and their potential for treating NAFLD, further dissection of the regulation of hepatic metabolic pathways by DPP4 is warranted (27–33). Here, we evaluated the role of hepatic DPP4 on incretin bioactivity within the portal vein (PV), hepatic glucose metabolism, and chronic liver disease progression in mice. We additionally examined circulating DPP4 levels and the mRNA abundances of sheddases in a mouse model of severe metabolic liver disease without obesity, phosphatidylethanolamine *N*-methyltransferase (*Pemt*^–/–^) mice. We also evaluated circulating DPP4 levels and substrates in a patient population undergoing treatment for HCV.

## Results

### Reduced HGP in high-fat, high-cholesterol–fed Dpp4^–/–^ mice is due to loss of hepatocyte-derived DPP4.

**Figure 1:**
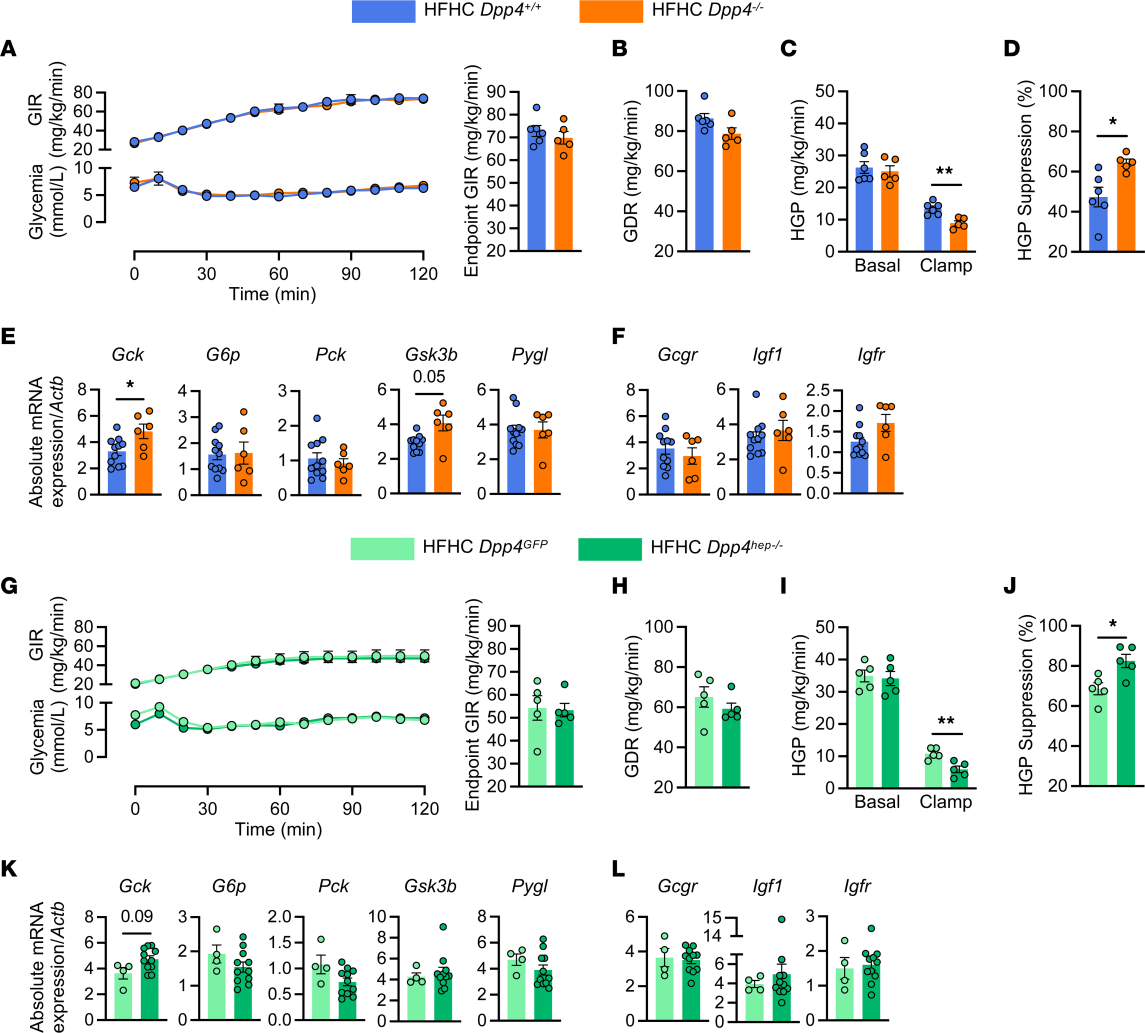
HGP is decreased in HFHC-fed *Dpp4^–/–^* and hepatocyte-specific *Dpp4*-knockout mice. (**A**) Time course of glucose infusion rates (GIRs) and plasma glucose and endpoint GIR during hyperinsulinemic-euglycemic clamp of *Dpp4^+/+^* (*n* = 6) and *Dpp4^–/–^* (*n* = 5) mice. (**B**) Glucose disposal rate (GDR), (**C**) basal and clamp HGP, and (**D**) percentage of HGP suppression under clamp conditions. Hepatic mRNA abundance (relative to *Actb*) for genes associated with (**E**) hepatic gluconeogenesis (*Gck*, *G6p*, *Pck*, *Gsk3*β, and *Pygl*) and (**F**) insulin signaling (*Gcgr*, *Igf1*, and *Igf1r*) in *Dpp4^+/+^* (*n* = 11) and *Dpp4^–/–^* (*n* = 7) mice. *Gck*, glucokinase; *G6p*, glucose-6-phosphatase; *Pck*, phosphoenolpyruvate carboxykinase; *Gsk3*β, glycogen synthase kinase 3β; *Pygl*, glycogen phosphorylase; *Gcgr*, glucagon receptor; *Igf1*, insulin-like growth factor 1; *Igf1r*, Igf1 receptor. (**G**) Time course of plasma glucose and glucose infusion rates, and endpoint GIR during hyperinsulinemic-euglycemic clamp of *Dpp4^GFP^* (*n* = 4) and *Dpp4^hep–/–^* (*n* = 5) mice. (**H**) GDR, (**I**) basal and clamp HGP, and (**J**) percentage of HGP suppression under clamp conditions. Hepatic mRNA abundance (relative to *Actb*) for genes associated with (**K**) hepatic gluconeogenesis (*Gck*, *G6p*, *Pck*, *Gsk3*β, and *Pygl*) and (**L**) insulin signaling (*Gcgr*, *Igf*, and *Igf1r*) in *Dpp4^GFP^* (*n* = 4) and *Dpp4^hep–/–^* (*n* = 11) mice. Data are presented as the mean ± SEM. Time-course data are analyzed by 2-way ANOVA with Tukey’s multiple comparisons post hoc test; remaining data analyzed by unpaired Student’s *t* test with Welch’s correction, *P = 0.01–0.05, **P = 0.001–0.01.

In patients with T2D, DPP4 inhibitors contribute to glucose homeostasis by decreasing hepatic gluconeogenesis (26, 34). To validate this effect in mice, we performed hyperinsulinemic-euglycemic clamps in *Dpp4^+/+^* (wild-type, WT) and *Dpp4^–/–^* (global *Dpp4* deletion) mice fed a high-fat, high-cholesterol (HFHC) diet for 12 weeks. Glucose infusion rates (GIRs, Figure 1A) and glucose disposal rates (GDRs, Figure 1B) were indistinguishable between *Dpp4^–/–^* mice and their WT littermate controls. Although basal levels of HGP were unchanged, insulin-stimulated HGP was significantly lower in HFHC-fed *Dpp4^–/–^* (Figure 1C), resulting in a significantly greater suppression of HGP by insulin in *Dpp4^–/–^* mice (Figure 1D). Body weight and fasting glucose levels were unchanged (Supplemental Figure 1, A and B; supplemental material available online with this article; https://doi.org/10.1172/jci.insight.154314DS1). Although the mRNA abundance of hepatic *Gck*, the enzyme that phosphorylates glucose to produce glucose-6-phosphate, was significantly upregulated in *Dpp4^–/–^* mice compared with livers of control mice (Figure 1E), transcript levels of the gluconeogenic enzymes, *Pck* and *G6p*, were unchanged (Figure 1E). mRNA levels of hepatic *Gsk3*β, a protein kinase that phosphorylates and inhibits glycogen synthase, were also elevated in *Dpp4^–/–^* mice compared with *Dpp4^+/+^* (Figure 1E). Hepatic mRNA expression of *Pygl*, *Gcgr*, *Igf1*, and *Igf1r* was indistinguishable between *Dpp4^–/–^* and *Dpp4^+/+^* mice (Figure 1, E and F).

To determine if the reduction in HGP in *Dpp4*^–/–^ mice was mediated through the actions of hepatocyte-derived DPP4, we then measured HGP in HFHC-fed, hepatocyte-specific *Dpp4*-knockout mice (*Dpp4^hep–/–^*). Similar to *Dpp4^–/–^* mice, HFHC-fed control mice (*Dpp4^GFP^*) and *Dpp4^hep–/–^* mice did not differ in GIR (Figure 1G) or GDR (Figure 1H) or in body weight or fasting glucose (Supplemental Figure 1, C and D). In addition, as seen in *Dpp4^–/–^* mice, basal levels of HGP were unchanged, and *Dpp4^hep–/–^* mice showed lower HGP under clamp conditions and greater insulin suppression of HGP (Figure 1, I and J). Akin to *Dpp4^–/–^* mice, mRNA expression of hepatic glucose utilization or gluconeogenic enzymes was unchanged except *Gck*, which trended (*P* = 0.09) upward in *Dpp4^hep–/–^* mice (Figure 1K). The gene expression of *Gcgr*, *Igf1*, and *Igf1r* was comparable between genotypes (Figure 1L).

### Deletion of Dpp4 in hepatocytes lowers portal DPP4 activity and increases portal concentrations of bioactive GLP-1 and GIP.

**Figure 2:**
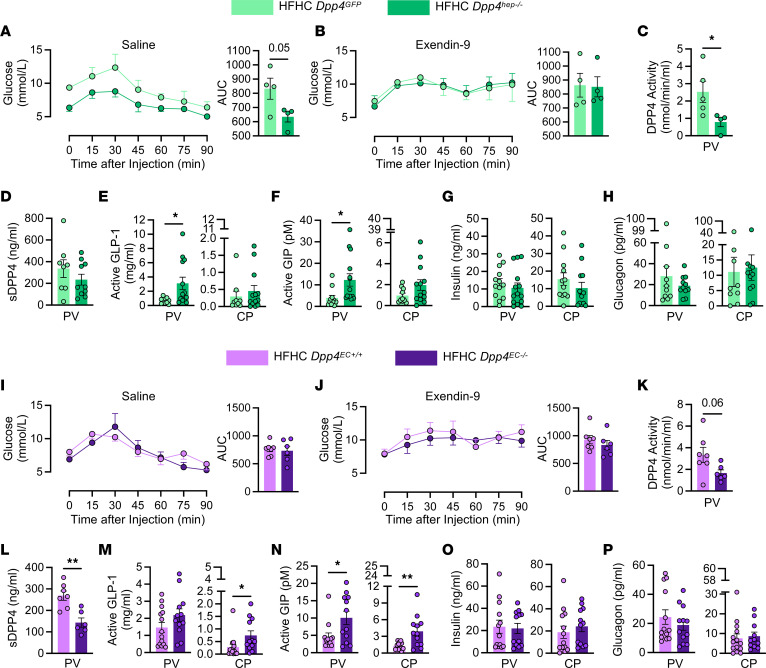
HGP is decreased in *Dpp4^hep–/–^* mice but not mice with deletion of *Dpp4* from Tie2^+^ endothelial cells via GLP-1– and GIP-dependent pathways. Blood glucose prior to and during a pyruvate tolerance test (PTT) following (**A**) saline or (**B**) exendin-9-39 i.p. injection and resulting AUC in *Dpp4^GFP^* (*n* = 4) and *Dpp4^hep–/–^* (*n* = 4) mice. (**C**) DPP4 activity, (**D**) soluble DPP4 concentration, (**E**) active GLP-1, (**F**) active GIP, (**G**) insulin, and (**H**) glucagon from the PV and CP 15 minutes after glucose bolus in *Dpp4^GFP^* (*n* = 4–12) and *Dpp4^hep–/–^* (*n* = 4–11) mice. Blood glucose prior to and during a PTT following (**I**) saline or (**J**) exendin-9-39 i.p. injection and resulting AUC in *Dpp4^EC+/+^* (*n* =7) and *Dpp4^EC–/–^* (*n* = 6) mice. (**K**) DPP4 activity, (**L**) soluble DPP4 concentration, (**M**) active GLP-1, (**N**) active GIP, (**O**) insulin, and (**P**) glucagon from the PV and CP 15 minutes after glucose bolus in *Dpp4^EC+/+^* (*n* = 7–13) and *Dpp4^EC–/–^* (*n* = 6–11) mice. Data are presented as the means ± SEM. Time-course data are analyzed by 2-way ANOVA with Tukey’s multiple comparisons post hoc test; remaining data analyzed by unpaired Student’s *t* test with Welch’s correction, *P = 0.01–0.05, **P = 0.001–0.01.

We evaluated glucose excursion after intraperitoneal injection of pyruvate to further evaluate the cell-specific roles of DPP4 in regulating HGP. Consistent with our previous work (22), *Dpp4^hep–/–^* mice had significantly reduced *Dpp4* mRNA expression in whole liver extracts (Supplemental Figure 2A); fasted DPP4 activity in plasma was decreased 50% in 20-week-old *Dpp4^GFP^* and *Dpp4^hep–/–^* mice fed an HFHC diet for 4 weeks (Supplemental Figure 2B); but DPP4 plasma concentration was unchanged (Supplemental Figure 2C). Accordant with the clamp data, *Dpp4^hep–/–^* mice had significantly reduced glucose excursion AUC after injection of pyruvate compared with littermate *Dpp4^GFP^* controls (Figure 2A). Next, we examined whether reduced HGP in *Dpp4^hep–/–^* mice is mediated by DPP4’s action on GLP-1 receptor (GLP-1R) signaling. The decrease in plasma glucose following pyruvate injection was abrogated with the administration of exendin-9-39, a compound that blocks signaling through the GLP-1R (35), 15 minutes before injection of pyruvate (Figure 2B). *Dpp4^hep–/–^* mice showed no change compared to *Dpp4^GFP^* in an arginine tolerance test, indicating that islet responsivity to acute depolarization was normal (Supplemental Figure 2D). In addition, in response to arginine, mice lacking hepatocyte *Dpp4* did not demonstrate any differences in blood glucose, active GLP-1, insulin, or glucagon (Supplemental Figure 2, E–G). GLP-1 has been reported to circulate through the portal circulation and the lymphatics to enter systemic circulation at the thoracic duct (36, 37). To evaluate how DPP4 in hepatocytes may influence the bioactivity of incretin hormones at each of these sites (i.e., portal or systemic concentration), we administered oral glucose to *Dpp4^hep–/–^* and control mice and sampled PV blood via cannulation followed by systemic blood 15 minutes later by cardiac puncture (CP). DPP4 activity but not protein level within the PV was significantly reduced in *Dpp4^hep–/–^* mice compared with *Dpp4^GFP^* mice (Figure 2, C and D). In addition, active GLP-1 and active GIP levels were elevated approximately 4-fold in the local portal circulation in *Dpp4^hep–/–^* mice compared with *Dpp4^GFP^* mice (Figure 2, E and F). On the other hand, no significant differences in active GLP-1 and GIP were noted in plasma isolated immediately in the same mice by CP (Figure 2, E and F). Despite the increase in circulating incretin, plasma insulin and glucagon levels were unchanged in local hepatic and systemic circulations (Figure 2, G and H).

To investigate the role of DPP4 in hepatocytes versus other sites that contribute to circulating DPP4, including TEK receptor tyrosine kinase–positive (Tie2^+^) endothelial cells (ECs) and immune cells, we used *Dpp4^EC–/–^* mice. Previously, we have reported that deletion of *Dpp4* from the Tie2^+^ ECs (*Dpp4^EC–/–^*) of high-fat–fed mice results in increased systemic concentrations of GLP-1 and improved glucose excursion but does not affect HGP (38). As expected, in contrast to *Dpp4^hep–/–^*, *Dpp4^EC–/–^* mice had no decrease in hepatic *Dpp4* mRNA expression (Supplemental Figure 2H), and, consistent with previous reports (38), systemic fasting plasma DPP4 activity and DPP4 protein levels (Supplemental Figure 2, I and J) were significantly decreased in *Dpp4^EC–/–^* mice compared with controls. Furthermore, a pyruvate tolerance test showed no change in HGP in HFHC-fed *Dpp4^EC+/+^* and *Dpp4^EC–/–^* mice previously administered saline or exendin-9-39 (Figure 2, I and J), consistent with our previous hyperglycemic-euglycemic clamp analyses (38).

To directly determine how DPP4 in these different tissue settings governs incretin bioactivity and HGP, we measured the portal concentrations of DPP4 and incretins and also examined pyruvate tolerance in HFHC-fed *Dpp4^EC+/+^* and *Dpp4^EC–/–^* mice. Although portal DPP4 activity was unchanged (Figure 2K), DPP4 protein levels in the PV were significantly decreased in *Dpp4^EC–/–^* mice (Figure 2L). Furthermore, levels of active GLP-1 and active GIP were unchanged in the portal circulation in *Dpp4^EC–/–^* mice, but systemic concentrations of incretins were increased 2.5-fold (GLP-1) or trended 2-fold higher (GIP) in samples isolated by CP (Figure 2, M and N). Levels of insulin and glucagon at either sampling site (Figure 2, O and P) were unaltered. Together, these results suggest that the reduction in HGP observed in HFHC-fed *Dpp4^–/–^* mice is driven by the loss of *Dpp4* in hepatocytes and not Tie2^+^ EC populations. Furthermore, this improved suppression of HGP is associated with elevated incretin concentrations within the PV, not the systemic circulation.

### Dyslipidemia and liver steatosis are unaffected by the genetic elimination of Dpp4 in aged, HFHC-fed mice.

**Figure 3:**
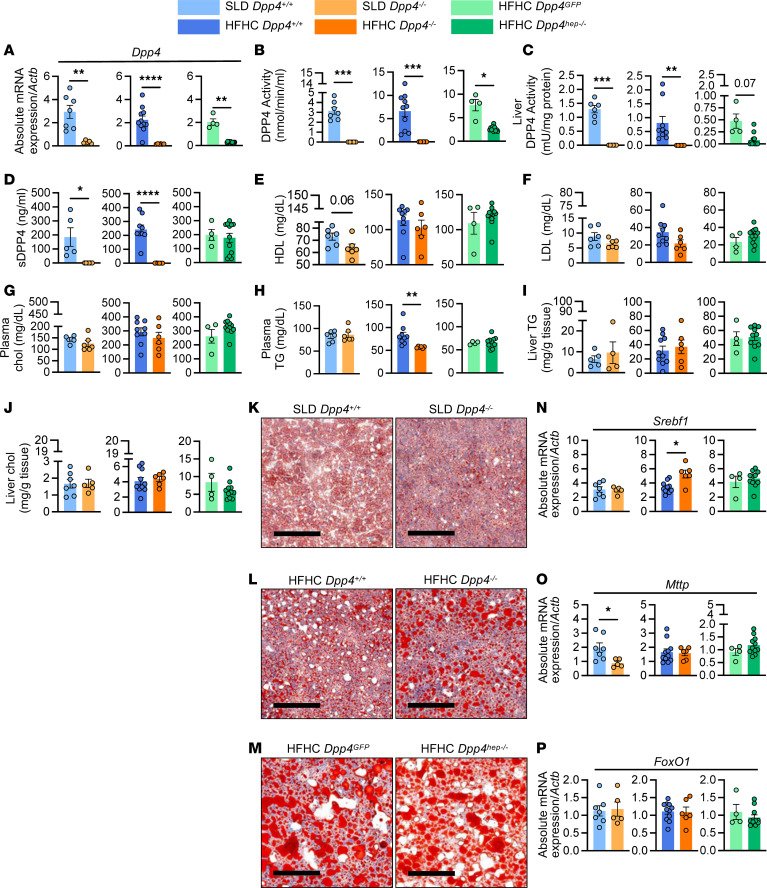
Plasma and liver lipid profiles remain largely unchanged between genotypes. (**A**) Hepatic *Dpp4* mRNA abundance (relative to *Actb*). (**B**) Plasma DPP4 activity. (**C**) Liver DPP4 activity. (**D**) Plasma DPP4 protein. (**E**) Plasma HDL, (**F**) LDL, (**G**) plasma cholesterol (chol), (**H**) plasma triglycerides (TG), (**I**) liver TG mass, and (**J**) total chol mass. Representative images of liver stained with Oil Red O in (**K**) SLD-fed *Dpp4^+/+^* (*n* = 5–7) and *Dpp4^–/–^* (*n* = 4–6) mice, (**L**) HFHC-fed *Dpp4^+/+^* (*n* = 9–11) and *Dpp4^–/–^* (*n* = 6) mice, and (**M**) HFHC-fed *Dpp4^GFP^* (*n* = 4) and *Dpp4^hep–/–^* (*n* = 10–11) mice (scale bar: 200 μm). Hepatic mRNA abundance (relative to *Actb*) of (**N**) *Srebf1*, (**O**) *Mttp*, and (**P**) *FoxO1*. Data are presented as the means ± SEM, analyzed by unpaired Student’s *t* test with Welch’s correction; *P = 0.01–0.05, **P = 0.001–0.01, ***P = 0.0001–0.001, and ****P < 0.0001.

Insulin resistance, de novo lipogenesis, and dysregulated blood glucose are key hallmarks in the progression of NAFLD as part of the multiple-hit hypothesis (39). To determine whether the reduction in HGP observed following the deletion of *Dpp4* from the whole animal or liver only can prevent NAFLD progression, we fed aged 6-month-old mice either a standard laboratory diet (SLD) or an HFHC diet for 24 weeks. In a small subset of aged mice (*n* = 2/group), we performed similar PV cannulations and CPs and observed trends consistent with those shown in Figure 2 (Supplemental Table 1). Analysis of *Dpp4* mRNA in whole liver extracts revealed elimination in all expected genotypes (Figure 3A). DPP4 activity in plasma and liver was absent from *Dpp4*^–/–^ on SLD and HFHC diet and reduced by 60% and 75% in *Dpp4^hep–/–^* mice compared with controls (Figure 3, B and C). Agreeing with our previous results (22), deletion of hepatocyte *Dpp4* led to sustained reductions in plasma and hepatic DPP4 activities, with little change in circulating DPP4 protein levels (Figure 3D). Deletion of hepatocyte-specific DPP4 in these aged mice did not affect glucose tolerance as only whole-body deletion of DPP4 increased systemic active GLP-1 and led to reduced blood glucose excursion during oral glucose tolerance test (Supplemental Figure 3, A and B), consistent with previous work (22, 23). Glycogen concentrations were unchanged in all settings (Supplemental Figure 3C). In both SLD-fed and HFHC-fed *Dpp4^–/–^* mice, lack of *Dpp4* did not affect HDL, LDL, or total cholesterol levels in plasma (Figure 3, E–G). However, fasting plasma triglycerides were significantly reduced in *Dpp4^–/–^* mice (Figure 3H). Biochemical measurement and histochemical analysis with Oil Red O staining in livers revealed no differences in neutral lipid concentrations between genotypes (Figure 3, I–M). Hepatic gene expression analysis also revealed that sterol regulatory element–binding transcription factor 1 (*Srebf1*) mRNA expression was significantly upregulated in HFHC-fed *Dpp4^–/–^* mice but unchanged in SLD-fed mice and HFHC-fed *Dpp4^hep–/–^* mice, compared with controls (Figure 3N). Microsomal triglyceride transfer protein (*Mttp*) mRNA expression was significantly decreased in SLD-fed *Dpp4^–/–^* mice compared with controls but unchanged in all genotypes under HFHC feeding (Figure 3O). In contrast, hepatic Forkhead box protein O1 (FoxO1) expression was unchanged between all genotypes under both diets (Figure 3P). Taken together, these data suggest that hepatic lipid accumulation is largely unaffected by whole-body or hepatocyte-specific elimination of *Dpp4* gene in aged, SLD- or HFHC-fed mice.

### Systemic, not hepatic, loss of Dpp4 in aged mice increases hepatic fibrosis.

**Figure 4:**
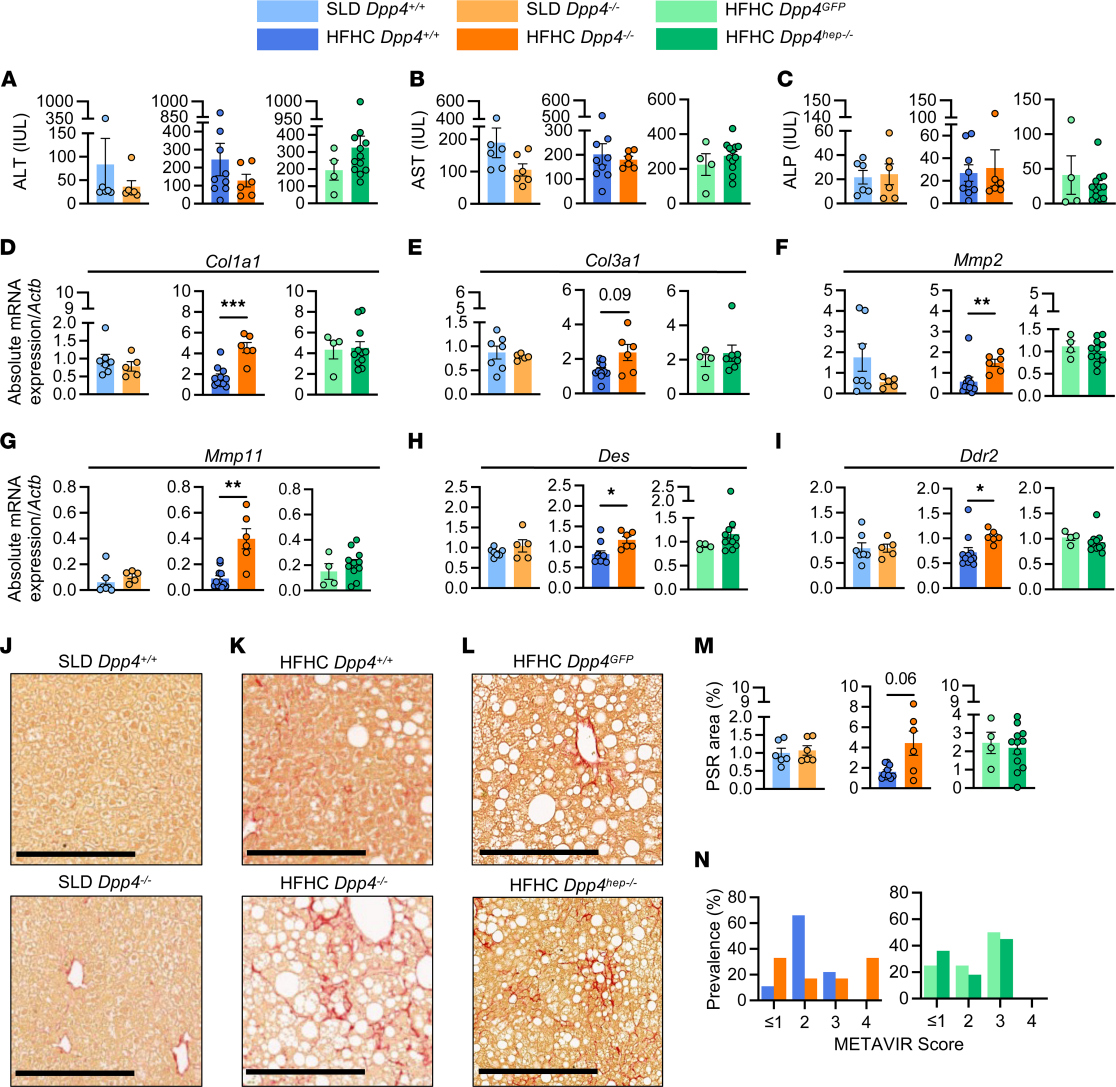
Fibrosis-related genes are upregulated in HFHC-fed *Dpp4^–/–^* but not *Dpp4^hep–/–^* mice. (**A**) Alanine aminotransferase (ALT), (**B**) aspartate transaminase (AST), and (**C**) alkaline phosphatase (ALP). Liver mRNA abundance (relative to *Actb*) of (**D**) *Col1a1*, (**E**) *Col3a1*, (**F**) *Mmp2*, (**G**) *Mmp11*, (**H**) *Des*, and (**I**) *Ddr2*. Representative images of liver stained with Picrosirius red (PSR) and quantitation of red-stained pixels in (**J**) SLD-fed *Dpp4^+/+^* (*n* = 6–7) and *Dpp4^–/–^* (*n* = 5–6) mice, (**K**) HFHC-fed *Dpp4^+/+^* (*n* = 9–11) and *Dpp4^–/–^* (*n* = 6) mice, and (**L**) HFHC-fed *Dpp4^GFP^* (*n* = 4) and *Dpp4^hep–/–^* (*n* = 11–12) mice (scale bar: 250 μm) and (**M**) quantitation of red-stained pixels. (**N**) Liver METAVIR score prevalence. Data are presented as the means ± SEM, analyzed by unpaired Student’s *t* test with Welch’s correction; *P = 0.01–0.05, **P = 0.001–0.01, and ***P = 0.0001–0.001.

Soluble, circulating DPP4 has been shown to be a marker of liver fibrosis (40). Thus, we examined if systemic and hepatic loss of *Dpp4* in mice affected liver fibrosis. Liver damage markers, such as alanine aminotransferase (ALT), aspartate transaminase (AST), and alkaline phosphatase, were not significantly different between all groups and their respective controls (Figure 4, A–C). Liver size normalized to tibia length was also unchanged (data not shown). To our surprise, mRNA levels of fibrosis markers, including *Col1a1*, *Col3a1*, *Mmp2*, *Mmp11*, *Des*, and *Ddr2*, were elevated in HFHC-fed *Dpp4^–/–^* mouse livers (Figure 4, D–I), suggesting worsening fibrosis in the *Dpp4^–/–^* mice. However, this elevated gene expression was not observed in *Dpp4^hep–/–^* mouse livers. No changes of expression were noted in fibrosis and hepatic stellate cell activation factors, *Mmp9*, *Gfap* and *Vim*, between any of the genotypes (Supplemental Figure 4, A–C) while both *Lrat* and *Pcdh7* were significantly reduced in SLD-fed *Dpp4^–/–^* mice compared with controls (Supplemental Figure 4, D and E). Consistent with gene expression, visualization of collagen with Picrosirius red staining revealed that HFHC-fed *Dpp4^–/–^* mice had elevated fibrotic area in the liver, whereas it was relatively unchanged in *Dpp4^GFP^* and *Dpp4^hep–/–^* mice (Figure 4, J–M). Supporting increased fibrosis, blinded meta-analysis of histological data in viral hepatitis (METAVIR) histopathological scoring of liver samples revealed a shift of 33% in *Dpp4^–/–^* mice to level 4 relative to littermate controls, while no *Dpp4^hep–/–^* mice were scored in this range (Figure 4N). Overall, these data suggest that systemic, not hepatic, loss of *Dpp4* increases liver fibrosis.

### Global, but not hepatic, loss of DPP4 increases expression of genes associated with adaptive immunity, inflammasome, and senescence-associated genes and pathways.

**Figure 5:**
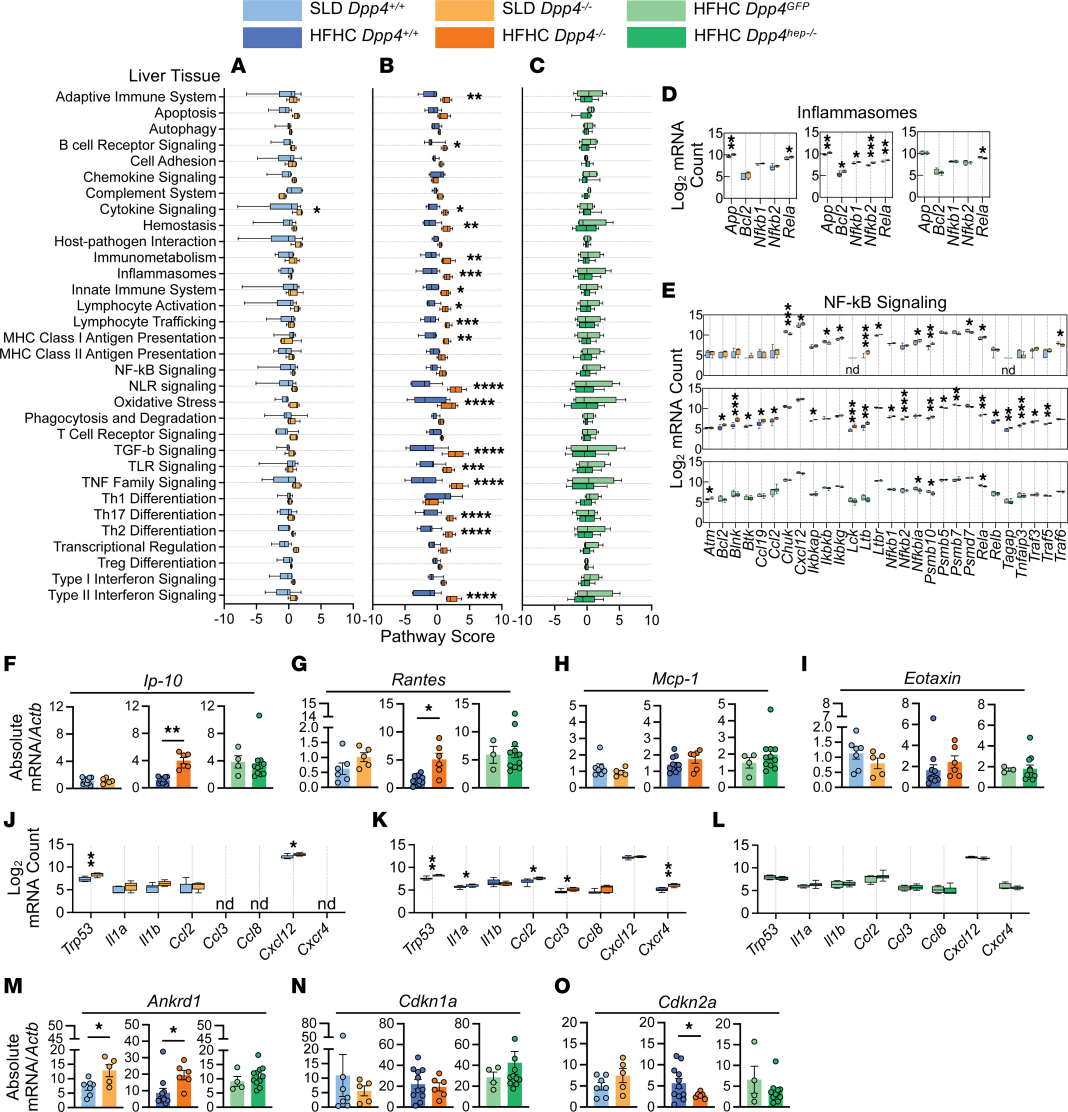
In whole liver tissue, immune-related genes and pathways are upregulated in HFHC-fed *Dpp4^–/–^* mice but unchanged in *Dpp4^hep–/–^* mice. Pathway scores of immunological pathways in liver tissue of (**A**) SLD-fed *Dpp4^+/+^* (*n* = 7) and *Dpp4^–/–^* (*n* = 5) mice, (**B**) HFHC-fed *Dpp4^+/+^* (*n* = 11) and *Dpp4^–/–^* (*n* = 6) mice, and (**C**) HFHC-fed *Dpp4^GFP^* (*n* = 4) and *Dpp4^hep–/–^* (*n* = 11) mice. Log_2_-normalized mRNA counts of genes associated with (**D**) inflammasome pathways and (**E**) NF-κB signaling pathways. Liver mRNA abundance (relative to *Actb*) of known DPP4 substrates and chemokines/cytokines of (**F**) *Ip-10*, (**G**) *Rantes*, (**H**) *Mcp-1*, and (**I**) *Eotaxin* in liver tissue. Log_2_-normalized mRNA counts of senescence-associated signaling phenotype (SASP) genes in liver tissue of (**J**) SLD-fed *Dpp4^+/+^* and *Dpp4^–/–^* mice, (**K**) HFHC-fed *Dpp4^+/+^* and *Dpp4^–/–^* mice, and (**L**) HFHC-fed *Dpp4^GFP^* and *Dpp4^hep–/–^* mice. Liver mRNA abundance (relative to *Actb*) of SASP genes (**M**) *Ankrd1*, (**N**) *Cdkn1a*, and (**O**) *Cdkn2a*. Box-and-whisker plots: box extends from the 25th to 75th percentiles; the whiskers go down to the smallest value and up to the largest. Data are presented as the means ± SEM, analyzed by unpaired Student’s *t* test with Welch’s correction, *P = 0.01–0.05, **P = 0.001–0.01, ***P = 0.0001–0.001, and ****P < 0.0001. nd, not detected.

To gain molecular insights into the inflammatory responses in the liver mediated by loss of *Dpp4*, we conducted NanoString mRNA analysis on liver tissue using an immunology panel of over 500 immune-related genes. All *Dpp4^hep–/–^* mice were confirmed by quantitative real-time PCR (qRT-PCR) with primers specific for the recombined *Dpp4* flox sites (Figure 3A), given the location of the flox deletion site toward the C-terminal end of the *Dpp4* transcript and modest reduction in gene expression detected by NanoString probes (Supplemental Figure 5A). Supervised hierarchical clustering analysis of differentially expressed genes in *Dpp4^–/–^* mice revealed a distinct cluster of genes that were upregulated in SLD- and HFHC-fed *Dpp4^–/–^* mice compared with *Dpp4^+/+^* and *Dpp4^GFP^* versus *Dpp4^hep–/–^* (Supplemental Figure 5, B and C). We identified differentially expressed genes that were distinct and overlapping among SLD-fed *Dpp4^–/–^*, HFHC-fed *Dpp4^–/–^*, and HFHC-fed *Dpp4^hep–/–^* livers (Supplemental Figure 5D). Pathway analysis in each comparison was performed, showing that only cytokine signaling in SLD-fed *Dpp4^–/–^* mice was significantly upregulated compared with *Dpp4^+/+^* mice (Figure 5A). Notably, 18 pathways, including adaptive and innate immune pathways, inflammasome, Toll-like receptor signaling, oxidative stress, and TGF-β signaling, were all significantly upregulated in HFHC-fed *Dpp4^–/–^* compared with *Dpp4^+/+^* mice (Figure 5B). Consistent with no difference in liver fibrosis (Figure 4H), all pathways were indistinguishable between HFHC-fed *Dpp4^GFP^* and *Dpp4^hep–/–^* mouse livers (Figure 5C), suggesting that hepatocyte DPP4 was not influencing the immunological response. We validated these results by immunostaining for the top differentially expressed transcript, Marco, in mice fed the HFHC diet. Consistent with the NanoString analysis, Marco staining was significantly reduced in the HFHC-fed *Dpp4^–/–^* mouse livers compared with controls (Supplemental Figure 6, A–G). Additionally, Marco expression in *Dpp4^GFP^* mice exhibited a spread of high- and low-expressing livers, which was recapitulated with immunostaining (Supplemental Figure 6, C, D, F, and G). When we probed gene expression within the inflammasome pathway, whose activation is a contributing factor in the initial progression of NAFLD (41), we found that all genes (*App*, *Bcl2*, *Nfkb1*, *Nfkb2*, and *Rela*) were significantly upregulated in HFHC-fed *Dpp4^–/–^* mice (Figure 5D). In contrast, only *App* and *Rela* were significantly upregulated in SLD-fed *Dpp4^–/–^* mice compared with controls, and *Rela* was significantly downregulated in *Dpp4^hep–/–^* mice (Figure 5D). Similarly, 19 of 28 NF-κB signaling pathway genes were significantly upregulated in HFHC-fed *Dpp4^–/–^* mice, whereas many genes were unchanged, or downregulated, in SLD-fed *Dpp4^–/–^* mice and HFHC-fed *Dpp4^hep–/–^* mice, compared with respective controls (Figure 5E). To further complement this analysis, we analyzed mRNA expression of known chemokine substrates of DPP4 in whole liver extracts. Consistent with its role in NAFLD progression (42), *Ip-10* (*Cxcl10*) gene expression was significantly upregulated in HFHC-fed *Dpp4^–/–^* mice compared with controls but unchanged in SLD-fed mice and HFHC-fed *Dpp4^hep–/–^* mice compared with controls (Figure 5F). Expression of the gene regulated on activation, T cell expressed, and secreted (*RANTES*; *Ccl5*), which is associated with severe liver fibrosis (43), was also significantly upregulated in HFHC-fed *Dpp4^–/–^* mice (Figure 5G), whereas no changes in *Mcp-1* (*Ccl2*) or *Eotaxin* (*Ccl11*) gene expression were noted (Figure 5, H and I). In contrast, gene expression of *Ip-10*, *RANTES*, *Mcp-1*, and *Eotaxin* were unchanged between HFHC-fed *Dpp4^GFP^* and *Dpp4^hep–/–^* mice (Figure 5, F–I).

Cellular senescence has been identified to be involved in the transition from liver steatosis to a more severe phenotype involving hepatocyte ballooning and elevated fibrosis (44). DPP4 has been identified on the surface of senescent cells, preferentially sensitizing them to cytotoxicity by NK cells (45). Therefore, we were prompted to evaluate gene expression of senescence-associated secretory phenotype (SASP) factors (46) in our models. Our liver NanoString analysis identified increased expression of *Trp53* in both SLD-fed and HFHC-fed, aged *Dpp4^–/–^* compared with their respective controls (Figure 5, J and K), which was unchanged in *Dpp4^hep–/–^* mice compared with *Dpp4^GFP^* mice (Figure 5L). We additionally measured genes associated with p53 signaling (47, 48). The mRNA level of *Ankrd1* was increased in SLD- and HFHC-fed *Dpp4^–/–^*, but unchanged in *Dpp4^hep–/–^*, compared with controls (Figure 5M). *Cdkn1a* expression was unchanged across both diets, and all genotypes (Figure 5N), while *Cdkn2a* was significantly decreased in HFHC-fed *Dpp4^–/–^* mice only (Figure 5O). When we analyzed protein levels of chemokine and cytokine SASPs, 8 weeks after starting the diet, CXCL1 levels were significantly increased in SLD-fed *Dpp4^–/–^* mice (Supplemental Figure 7A), while IL-6 was significantly increased in HFHC-fed *Dpp4^–/–^* mice (Supplemental Figure 7B). Other plasma cytokines, including IL-1β, IFN-γ, IL-10, and IL-2, were unchanged (Supplemental Figure 7, C–H). However, in HFHC-fed mice, IL-4 was significantly decreased in *Dpp4^–/–^* mice compared with controls (Supplemental Figure 7I), while it was increased in *Dpp4^hep–/–^* as was IL-5 at both 8 weeks and endpoint (Supplemental Figure 7, I and G). Few other significant changes were noted in plasma or within liver tissue at endpoint (Supplemental Figure 7, J–Y).

### Immune-related genes are upregulated in F4/80^+^ cells of SLD-fed Dpp4^–/–^ mice, but HFHC feeding reduces DPP4 expression in F4/80^+^ cells.

**Figure 6:**
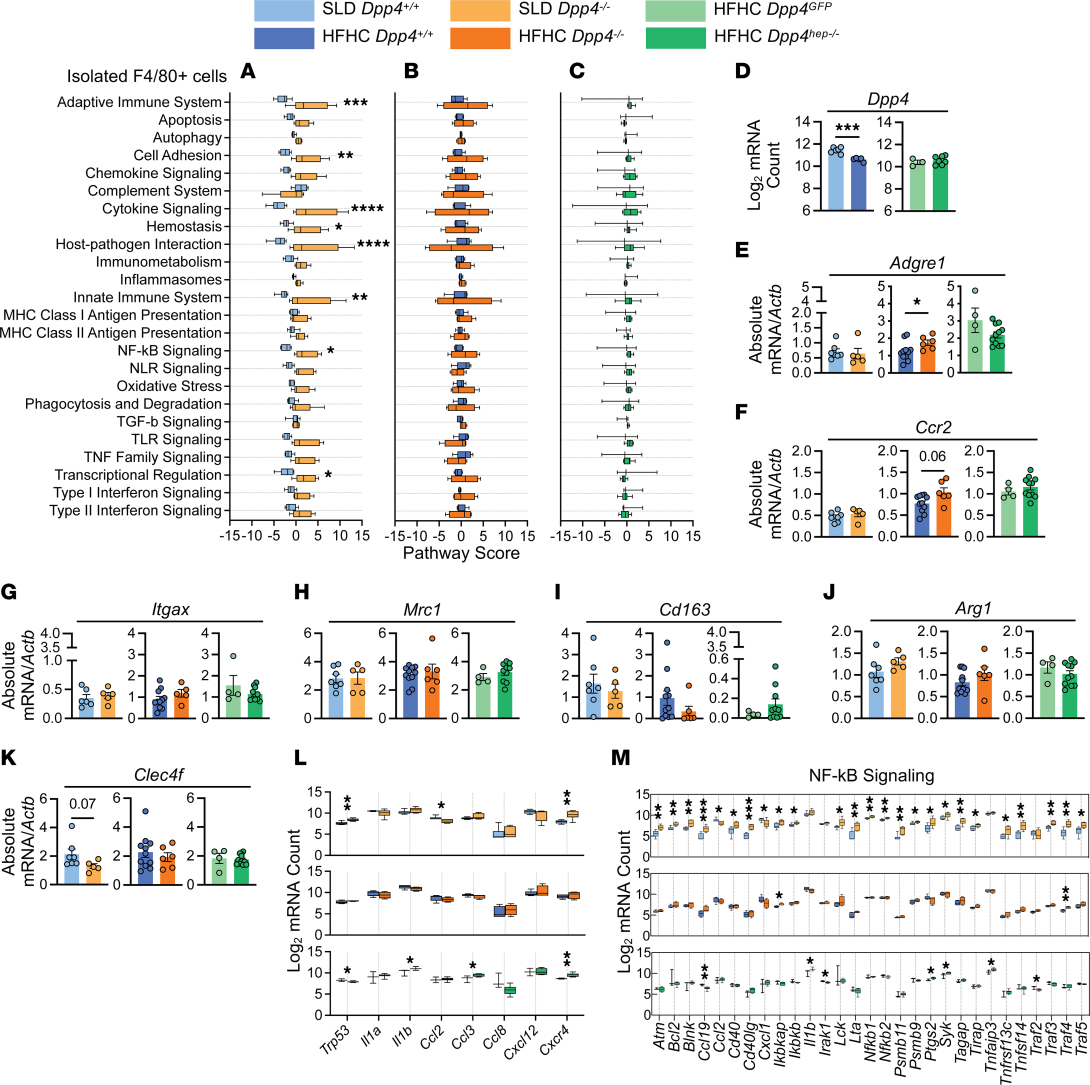
In hepatic F4/80^+^ cells, immune-related genes and pathways are upregulated in SLD-fed *Dpp4^–/–^* mice but unchanged in HFHC-fed *Dpp4^–/–^* and *Dpp4^hep–/–^* mice. Pathway scores of immunological pathways in F4/80^+^ cells isolated from liver tissue of (**A**) SLD-fed *Dpp4^+/+^* (*n* = 6) and *Dpp4^–/–^* (*n* = 6) mice, (**B**) HFHC-fed *Dpp4^+/+^* (*n* = 5) and *Dpp4^–/–^* (*n* = 4) mice, and (**C**) HFHC-fed *Dpp4^GFP^* (*n* = 3) and *Dpp4^hep–/–^* (*n* = 7) mice. (**D**) Log_2_-normalized mRNA count of *Dpp4* in F4/80^+^ cells isolated from liver. Liver mRNA abundance (relative to *Actb*) of (**E**) *Adgre1* and (**F**) *Ccr2*. Liver mRNA abundance (relative to *Actb*) of (**G**) *Itgax*, (**H**) *Mrc1*, (**I**) *Cd163*, (**J**) *Arg1*, and (**K**) *Clec4f*. (**L**) Log_2_-normalized mRNA counts of senescence-associated signaling phenotype (SASP) genes in F4/80^+^ cells. Log_2_-normalized mRNA counts of genes associated with (**M**) NF-κB signaling pathway in F4/80^+^ cells in SLD-fed *Dpp4^+/+^* (*n* = 7) and *Dpp4^–/–^* (*n* = 5) mice, HFHC-fed *Dpp4^+/+^* (*n* = 11) and *Dpp4^–/–^* (*n* = 5–6) mice, and HFHC-fed *Dpp4^GFP^* (*n* = 4) and *Dpp4^hep–/–^* (*n* = 11) mice. Box-and-whisker plots: box extends from the 25th to 75th percentiles; the whiskers go down to the smallest value and up to the largest. Data are presented as the means ± SEM, analyzed by unpaired Student’s *t* test with Welch’s correction; *P = 0.01–0.05, **P = 0.001–0.01, ***P = 0.0001–0.001, and ****P < 0.0001.

Roles of both liver-resident macrophages and recruited monocyte-derived macrophages in NAFLD progression (49) and liver fibrosis (50) have been established. Additionally, DPP4 is known to be upregulated when macrophages are polarized with proinflammatory stimuli and implicated in macrophage polarization and activation to mediate inflammation (51). We therefore probed if liver-resident macrophages were critical in driving the increased inflammation in livers of mice with global *Dpp4* deletion. We isolated F4/80^+^ cells from the liver and conducted NanoString mRNA analysis using the same immunology panel described above. Surprisingly, a large cluster of immune-related genes were upregulated in F4/80^+^ cells of SLD-fed *Dpp4^–/–^* mice compared with controls. However, these same genes were not differentially expressed in HFHC-fed *Dpp4^–/–^* mice (Supplemental Figure 8, A and C) whereas in HFHC-fed *Dpp4^hep–/–^* mice, 2 distinct clusters were revealed to be significantly altered compared with controls (Supplemental Figure 8, B and C). Pathway analysis revealed significant increases in NF-κB signaling, adaptive and innate immune system, and cytokine signaling (Figure 6A) in SLD-fed *Dpp4^–/–^* mice compared with controls. However, no pathways were significantly altered in HFHC-fed *Dpp4^–/–^* (Figure 6B) and *Dpp4^hep–/–^* mice (Figure 6C). In the isolated F4/80^+^ cells, unexpectedly, *Dpp4* was downregulated in HFHC-fed *Dpp4^+/+^* mice compared with SLD-fed *Dpp4^+/+^* mice. Its expression was unchanged between HFHC-fed *Dpp4^GFP^* and *Dpp4^hep–/–^* mice, verifying deletion was restricted to hepatocytes (Figure 6D). To further understand potential differences in F4/80^+^ cells’ composition within the liver, we assessed differences in the abundance of transcripts associated with characterized populations. We found *Adgre1* was upregulated in HFHC-fed *Dpp4^–/–^* mice (Figure 6E), and *Ccr2* trended toward increase in HFHC-fed *Dpp4^–/–^* mice compared with controls. These markers remained unchanged in SLD-fed *Dpp4^–/–^* and *Dpp4^hep–/–^* mice versus their respective controls (Figure 6F). However, no differences in F4/80 immunostaining were noted (Supplemental Figure 9, A–E) between the HFHC-fed groups. Additionally, no changes were observed in macrophage polarization and population markers, *Itgax* (Figure 6G), *Mrc1* (Figure 6H), *Cd163* (Figure 6I), *Arg1* (Figure 6J), and *Clec4f* (CLC4F) (Figure 6K), between genotypes and their respective controls. Consistent with these results, CLEC4F immunostaining revealed no differences between HFHC-fed groups (Supplemental Figure 9, A–D and F). *Trp53* was upregulated in SLD-fed *Dpp4^–/–^* and downregulated in HFHC-fed *Dpp4^hep–/–^*, while *Cxcr4* was upregulated in both (Figure 6L). SLD-fed *Dpp4^–/–^* mice had significant changes in many components of the NF-κB signaling pathway, but few of these patterns were observed in HFHC-fed *Dpp4^–/–^* or *Dpp4^hep–/–^* mice (Figure 6M). These data reveal an unexpected, complex relationship between DPP4 and liver-resident F4/80^+^ cells’ immunological profiles associated with diet composition.

### Liver-specific insults affect circulating DPP4 protein concentrations.

Given its strong correlation with adipose tissue accumulation and obesity in both humans and mice, soluble, circulating DPP4 was initially characterized as an adipokine (13, 22). Recent studies with adipocyte-specific targeting of DPP4 have determined that although adipocytes shed a small amount of DPP4 (22, 52), hepatocytes account for the significant elevation in DPP4 observed in high-fat diet feeding and metabolic dysregulation (22, 24). Further, liver *DPP4* expression is elevated in NAFLD (20, 21). Comprehensive studies in cultured hepatocytes have determined that a combination of leptin and palmitic acid stimulates a 6-fold increase in *Dpp4* mRNA expression (53). To test the necessity of adiposity and peripheral insulin resistance in vivo for elevated enzymatically active, circulating DPP4, we assessed plasma DPP4 activity in HFHC-fed *Pemt^–/–^* mice, which are a lean model of hepatomegaly and hepatic steatosis due to disruption in de novo synthesis of choline (54) (Supplemental Figure 10A). *Pemt^–/–^* mice do not develop obesity with high-fat feeding, retain insulin sensitivity, and have lower leptin concentrations compared with littermate controls (54, 55). Notably, systemic DPP4 activity was increased 4-fold relative to controls (Supplemental Figure 10B), suggesting dysregulation of hepatic lipid pathways is related to increased DPP4 activity, independent of the development of obesity. Unexpectedly, *Dpp4* mRNA level in the liver was unchanged (Supplemental Figure 10C). However, mRNA expression of candidate sheddases was significantly increased, including *Mmp9*, *Mmp2*, and *Adamst*, but not *Adam17* (Supplemental Figure 10, D–G).

**Figure 7:**
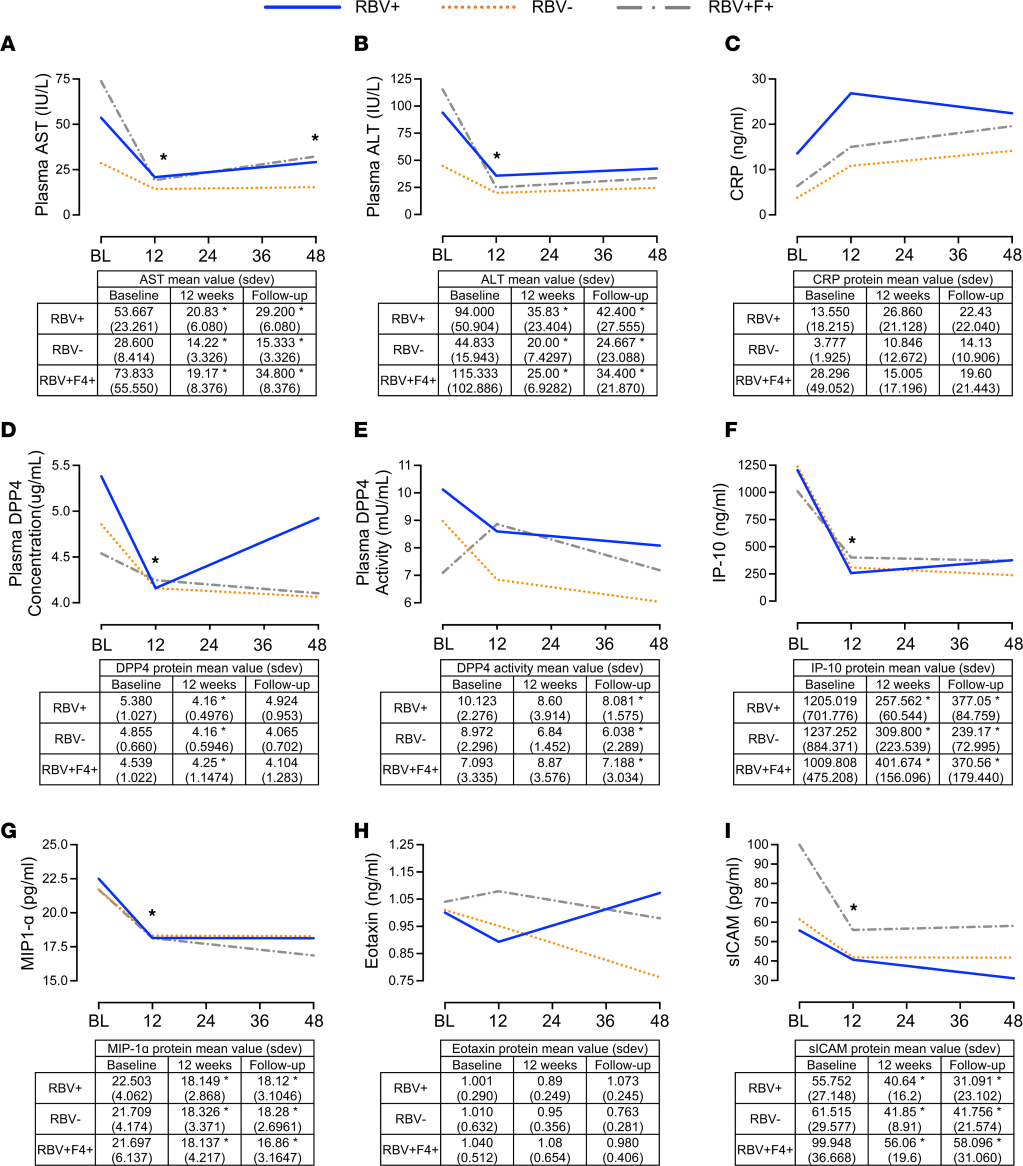
Independent of metabolic parameters, DPP4 concentration and activity decrease with HCV clearance and viral treatment. Changes in plasma (**A**) AST, (**B**) ALT, and (**C**) C-reactive protein (CRP) levels; (**D**) DPP4 concentration; (**E**) DPP4 activity; and concentration of cytokines and chemokines that are known substrates of DPP4, including (**F**) IP-10, (**G**) MIP-1α, (**H**) Eotaxin, and (**I**) sICAM-1, in patients (*n* = 5–6) with HOMA-IR > 2 treated for HCV. Time points: BL, baseline; week 12, end of treatment; follow-up, 24 to 48 weeks after treatment. Groups: RBV^+^, HCV treatment containing ribavirin in participants without cirrhosis; RBV^−^, ribavirin-free HCV treatment in participants without cirrhosis; RBV^+^F4^+^, HCV treatment containing ribavirin in participants with cirrhosis. To remove the variance in trait values attributed to sex and age differences, a linear regression model compared the retrieved residuals (adjusted trait values) using unpaired Student’s *t* test. Data are presented as the mean ± SD, and *P < 0.05.

In addition to steatosis, other liver-specific insults associated with elevated circulating DPP4 include HCV (56). Chronic HCV’s association with metabolic disease has been established (57). We have recently shown that in patients with HCV treated with paritaprevir/ritonavir/ombitasvir/dasabuvir (PrOD), with or without ribavirin, fasting glucose, insulin, and Homeostatic Model Assessment of Insulin Resistance (HOMA-IR) are unchanged during treatment and follow-up after treatment (58). We now sought to investigate whether elevated DPP4 levels are reversed following successful therapy for viral clearance in patients with metabolic dysfunction (HOMA-IR > 2) that persists through the treatment and follow-up period. Plasma AST (Figure 7A) and ALT (Figure 7B) significantly decreased in all treatment groups and were maintained at decreased levels during follow-up. Not surprisingly, given the lack of change in metabolic disease parameters, high variability and no significant changes were observed in C-reactive protein concentrations (Figure 7C). Consistent with other studies utilizing IFN-α treatment (59, 60), DPP4 concentration in plasma significantly decreased within all PrOD regimens from baseline to 12 weeks posttreatment (Figure 7D). In comparison, DPP4 activity substantially decreased with ribavirin treatment from baseline to follow-up in ribavirin-treated patients (Figure 7E). A similar trend to DPP4 protein was observed with known DPP4 substrates IP-10 (Figure 7F) and macrophage inflammatory protein 1α (MIP-1α) (Figure 7G). However, Eotaxin, another substrate, was unaffected (Figure 7H). Soluble intracellular adhesion molecule 1 (sICAM-1) was significantly decreased with treatment (Figure 7I). Taken together, DPP4 concentration and activity decrease with HCV treatment and viral clearance. However, this occurs independently of changes in metabolic parameters.

## Discussion

Potentiation of GLP-1 action through receptor agonists has demonstrated efficacy in treating metabolic disease (61, 62); however, our knowledge of the effects of DPP4 elimination to potentiate endogenous GLP-1 action within the context of chronic liver disease progression is limited. The present data demonstrate that eliminating hepatocyte DPP4 in HFHC-fed mice decreased DPP4 activity and increased intact incretins in the portal circulation and reduced HGP. These data are also consistent with patients in which DPP4 inhibitors (DPP4is) decrease HGP and are associated with reductions in glucagon (26, 34). In contrast, in HFHC-fed *Dpp4^EC–/–^*, we report increased levels of active GLP-1 in the systemic circulation and no effect on HGP as assessed by hyperinsulinemic-euglycemic clamp (38).

Studies in mice have also demonstrated that intact GLP-1R signaling within the portal circulation is integral to glucose sensing (63). This is interesting given that postprandial GLP-1 levels measured in the lymph are 5–6 times higher relative to sampling performed in portal plasma (36). Consistent with our data identifying different regulation of incretin bioactivity and modulation of glucose metabolism with DPP4 in hepatocytes versus Tie2^+^ cells, recent reports have determined that elevated levels of active portal GLP-1 are disconnected from the classic definition of the incretin effect as increased portal circulation of GLP-1 does not potentiate nutrient-stimulated insulin secretion (64, 65). In studies performed in rats, samples taken 20 minutes following a high-fat diet meal showed elevated GLP-1 in the lymph collected from the mesenteric lymph duct rather than the PV, while no difference was observed after a low-fat meal (66). Circulating DPP4 activity is lower in lymph than in plasma (36, 66). However, lymphatic ECs have been reported to express DPP4, and modulation of levels with siRNA affects migration and function (67). Therefore, our current study is consistent with a model where, during HFHC diet–induced metabolic dysregulation, the deletion of *Dpp4* within Tie2^+^ cells increases the abundance of GLP-1 delivered to the systemic circulation, enabling the incretin effect, and improves oral glucose tolerance but does not affect HGP. This is in contrast to *Dpp4* in hepatocytes, which when deleted in HFHC-fed mice, increases GLP-1 bioactivity within the portal circulation and improves insulin-mediated suppression of HGP. Our data align with results from *Hif1α^hep–/–^* mice, demonstrating that elevation in DPP4 through activation of hepatocyte HIF-1α reduces active GLP-1 in the portal circulation (53). Additionally, Baumeier et al. (25) demonstrated that hepatic *Dpp4* overexpression results in decreased active, glucose-stimulated GLP-1 in the vena cava after liver passage. Recent studies have documented that GLP-1R engagement in the portal circulation is reduced under high-fat diet–feeding conditions (68), suggesting together with our data that multiple mechanisms converge to control GLP-1 action in the hepatic portal circulation, which may contribute to glucose dysregulation in mice upon high-fat feeding.

Elevated concentrations of plasma DPP4 are associated with liver disease severity and fibrosis (69). Consistent with this, hepatocyte-specific overexpression of DPP4 in mice results in increased hepatic steatosis, liver enzymes, and markers of inflammation (25). In the current study, we report DPP4 was elevated 4-fold in *Pemt^–/–^* mice, which have both hepatomegaly and nonalcoholic steatohepatitis (54), demonstrating that in addition to obesity, liver-specific insults can induce the release of DPP4. Surprisingly, however, complete genetic elimination of *Dpp4* or hepatocyte-specific elimination in aged mice resulted in no changes in liver enzymes and the degree of steatosis. Consistent with our results, using liver-specific knockdown of DPP4 via therapeutic siRNA, in obese and diabetic *db/db* mice, no effect on liver enzymes or glucose tolerance is observed (23). Both Varin et al. and Ghorpade et al. reported modest impact on the liver with long-term targeting strategies (22, 24), suggesting that acute treatments targeting DPP4 more readily influence mouse lipid metabolism than long-term deletion of DPP4, which may be prone to metabolic adaptation.

In mice, DPP4is decrease liver fibrosis (70, 71); however, this was not recapitulated by genetically eliminating *Dpp4* as Picrosirius red staining revealed a trend toward increased fibrosis, and expression of *Col1a1* and *Col3a1* was increased in *Dpp4^–/–^* mice. Chronic liver inflammation and immune reactions often precede fibrosis (72); therefore, we assessed mRNA expression of immune-related genes. Under HFHC-fed conditions, livers of *Dpp4^–/–^* mice but not *Dpp4^hep–/–^* mice exhibited upregulation of transcripts associated with the inflammasome and markers of NF-κB signaling, pathways characterized to be activated in NAFLD (73, 74). This was surprising given that DPP4is have been reported to suppress NF-κB activation (75, 76). These results support important differences obtained by enzymatic inhibition versus complete deletion of the DPP4 protein.

Cellular senescence has been proposed as a key factor in NAFLD progression (77). Further, DPP4 has been identified as a surface protein that is enriched in senescent cells (45), and modulation of DPP4 in presenescent WI-38 cells (45) or in vascular endothelial cells (78) reduces markers of senescence. Additionally, senescence induced by glucocorticoid treatment, known to increase DPP4 transcriptionally (79), can be modulated by inhibition of DPP4 activity (80). In our study, lifelong deletion of DPP4 in aged mice led to upregulation of *Trp53*, a component of the senescent machinery (81). Additionally, the expression of *Ankrd1*, *Il1a*, *Ccl2*, and *Ccl3* was increased in HFHC-fed *Dpp4^–/–^* mice. A molecular link between p53 and DPP4 has been established in which p53 antagonizes ferroptosis by blocking DPP4 activity (82). However, the mechanistic link between DPP4, p53, cellular senescence, and liver fibrosis could not be deduced from this study and warrants further investigation.

Elevated DPP4 is also observed in other chronic liver diseases, such as HCV, in addition to NAFLD (15). Consistent with data from patients who received IFN treatment (59), plasma DPP4 concentrations, liver enzymes, and cytokine levels were reduced after resolution of infection with direct-acting antiviral therapy with or without ribavirin. DPP4 activity varied between treatment groups at baseline but overall remained decreased from baseline at follow-up in all. Additionally, plasma AST, ALT, IP-10, MIP-1α, and sICAM-1 all decreased with treatment. However, these changes were not associated with changes in glucose regulation (58).

We acknowledge several shortcomings to our study, including that the effects of exendin-9-39 cannot exclude the potential for signaling of glucagon through the GLP-1R (83–86), in addition to active GLP-1 (87). Additionally, while mice remained unrestrained during the hyperinsulinemic-euglycemic clamps, they were sampled by the tail vein, which may contribute differential physiological responses to stress across genotypes. In addition, many of our inferences are dependent on transcript levels rather than direct protein quantitation due to the availability and reliability of relevant antibodies.

In summary, we have identified hepatocyte-derived *Dpp4* as a key factor in regulating the bioactivity of GLP-1 in the PV and extended these findings to demonstrate that its elimination results in a reduction in HGP. However, despite the elevation in GLP-1 and improvements in hepatic insulin sensitivity, we demonstrate a disconnect as markers of lipid metabolism, fibrosis, and inflammation were unchanged or worsened in mice.

## Methods

### Animals.

All studies were performed according to protocols approved by the University of Ottawa Animal Care Committee and in accordance with guidelines of the Canadian Council on Animal Care. Male mice were housed under a 12-hour light/12-hour dark cycle and maintained on SLD (Harlan Teklad) or HFHC diet (TD.88137, Envigo-Teklad Custom Diets). Whole-body *Dpp4^–/–^* mice, on a C57BL/6 background, have been described (38, 88). To generate *Dpp4^hep–/–^* mice, *Dpp4^fl/fl^* adult mice, provided by Merck Research Laboratories (38), were i.v. injected with 1.5 × 10^11^ genome copies per mouse of AAV8.TBG.pi.egfp.wpre.bgh (AAV-GFP; control virus, 105535-AAV8, *Dpp4^GFP^*) or AAV8.TBG.PI.CRE.rBG (AAV-Cre; 107787-AAV8, *Dpp4^hep–/–^*) prior to the onset of HFHC diet feeding. Both AAV constructs were obtained from the University of Pennsylvania Vector Core Lab as a gift from James M. Wilson (Perelman School of Medicine, University of Pennsylvania, Philadelphia, Pennsylvania, USA). B6.Cg-Tg(Tek-cre) 1Ywa/J mice were obtained from The Jackson Laboratory (Strain 008863, RRID: IMSR_JAX:008863) and bred with *Dpp4^fl/fl^* to generate Dpp4^EC–/–^ mice. Experiments in *Pemt^+/+^* and *Pemt*^−*/*−^ mice, provided by LKS Centre for Health Research Innovation (89), were approved by the University of Alberta’s Institutional Animal Care Committee. Mice were fed an HFHC diet (F3282; Bio-Serv) for 3 weeks and fasted for 12 hours before sacrifice and tissue collection. Hyperinsulinemic-euglycemic clamps were performed in 12-week-old *Dpp4^+/+^*, *Dpp4^–/–^*, *Dpp4^fl/fl^*, and *Dpp4^hep–/–^* mice subjected to 12–16 weeks of HFHC diet. Experiments were performed in 18- to 20-week-old mice after 5 weeks of HFHC diet to validate our HGP results. Last, to assess NAFLD progression, experiments were performed in 16- to 28-week-old mice fed an HFHC diet for 24 weeks, with sacrifice and tissue collection at 40–52 weeks. Eight mice died prematurely (1 *Dpp4^hep–/–^*, 2 *Dpp4^GFP^*, 1 *Dpp4*^–/–^, and 4 WT mice), and 1 *Dpp4^GFP^* mouse was omitted from analysis due to the presence of a very enlarged spleen. Aged, HFHC-fed *Dpp4^hep–/–^* and *Dpp4^GFP^* mice were assessed for portal hormone concentrations (*n* = 2), with averages presented in Supplemental Table 1. Mice were genotyped from genomic DNA (gDNA) isolated from tail samples, and DNA recombination was confirmed in gDNA isolated from the liver. After DNA extraction and amplification, the PCR product was loaded on 1% agarose gels (UltraPure Agarose, Invitrogen, Thermo Fisher Scientific), then separated for 30 minutes at 150 V (Owl Easycast B2 Separating System), and the bands were visualized under a blue light transilluminator (UVP GelDoc-It2 Imager).

### Hyperinsulinemic-euglycemic clamp.

Hyperinsulinemic-euglycemic clamps were performed as previously described (90). Briefly, a catheter was surgically placed into the right jugular vein, and mice were allowed to recover for 5 days. All mice regained their presurgical weights following surgery. The catheter was made accessible through an adaptor port implanted in the dorsal subscapular region. On the day of the clamp, mice were fasted for 5 hours and then infused with d-[3-3H]-glucose (PerkinElmer) for 1 hour to evaluate basal glucose disposal. Human insulin (10 mU/kg/min, NovoRapid, Novo Nordisk) infusate containing d-[3-3H]-glucose was then administered, and blood glucose levels were titrated with 50% dextrose to achieve and maintain euglycemia. Mice were not physically restrained and were free to move around their cage, with blood samples taken via the tail vein. Basal and clamped rates of glucose disposal and HGP were calculated as described (90). All tissues were rapidly dissected, snap-frozen in liquid nitrogen, and stored at −80°C for later analyses.

### PV and CP cannulation.

Mice were given an oral bolus of glucose (2 g/kg) and subsequently anesthetized with 4% isoflurane. Once fully unconscious, the abdomen was disinfected, and the mouse was placed on a heated surgical platform. A sagittal incision was made through the skin and fascia of the lower abdomen. A lateral transverse incision was made through the muscular layer to expose the abdominal contents. The PV was gently exposed by moving the intestines laterally toward the left body wall. Once exposed, the PV was cannulated with a 20-gauge butterfly needle 15 minutes after the glucose bolus, and the needle was withdrawn to collect blood. Approximately 1 minute later, the beating heart was exposed by cutting through the diaphragm and thorax. An 18-gauge needle was used to collect blood from the right ventricle. Blood was aliquoted into 2 EDTA-coated capillary microvette tubes, one with 10% TED (5,000 KIU/mL Trasylol, 1.2 mg/mL EDTA, and 0.1 nmol/L Diprotin A) (vol/vol) and one without. Plasma was isolated after centrifugation (13,523*g*, 10 minutes, 4°C) and stored at −80°C for later analyses.

### Pyruvate, arginine, and glucose tolerance tests.

After a 16-hour fast, mice were intraperitoneally injected with either saline or exendin-9-39 (24 nmol/kg body weight; ref. 91; Bachem), 15 minutes prior to injection, with 2 g/kg body weight pyruvate in sterile 0.9% saline. After a 4-hour fast, mice were intraperitoneally injected with 2 g/kg body weight arginine in sterile 0.9% saline. For glucose tolerance tests, mice were fasted for 5 hours and given glucose in PBS (2 g/kg body weight) in sterile 0.9% saline. Blood for glucose measurements (Glucometer, MediCure Canada) was obtained from the tail vein before pyruvate injection and at 15, 30, 45, 60, and 90 minutes after pyruvate injection, or before arginine injection and at 15 and 30 minutes after arginine injection.

### Blood and tissue collection.

All blood samples were collected in EDTA-coated capillary microvette tubes, and plasma was isolated after centrifugation (13,523*g*, 10 minutes, 4°C). During metabolic tolerance tests, blood was taken via tail vein. For terminal studies, mice were sacrificed by CO_2_ inhalation, and blood was obtained by CP. For measurement of plasma active GLP-1 (Meso Scale Diagnostics) and active GIP (Crystal Chem), blood was mixed with 10% TED (vol/vol) and plasma stored at –80°C until further analysis. Plasma insulin (Alpco Diagnostics) and glucagon (Crystal Chem) levels were determined as per manufacturer’s instructions. Analysis for plasma ALT, AST, alkaline phosphatase, TG, cholesterol, LDL, and HDL was performed by the Pathology core at The Centre for Phenogenomics. The Beckman Coulter AU480 clinical chemistry analyzer was used in combination with appropriate reagents (ALT, AST, TG, cholesterol, LDL, and HDL), calibrators (Beckman Coulter Lyophilized Chemistry Calibrator levels 1 and 2), and quality control materials (Bio-Rad Liquid Assayed Multiqual levels 1 and 3). DPP4 activity was assessed using fluorometric assay (substrate: 10 mM H-Gly-Pro-AMC HBr, Bachem catalog I-1225; standard: AMC, Bachem catalog Q-1025). DPP4 protein level was measured using DPPIV/CD26 DuoSet ELISA kit (DY954; R&D Systems, Bio-Techne) following the manufacturer’s instructions.

### Picrosirius red and Oil Red O staining.

Liver tissue was fixed in 4% paraformaldehyde (PFA) and routinely processed for paraffin-embedding and cross-sectioned to obtain 5 μm–thick sections. The slides were incubated with a 0.1% Picrosirius red solution and mounted with Permount Mounting Medium (Thermo Fisher Scientific). Collagen accumulation was determined by the number of red-stained pixels using ImageJ (NIH). Accumulation of fat droplets in the liver was visualized using Lipid (Oil Red O) Staining Kit as per manufacturer protocol (BioVision). A pathologist assessed the Picrosirius red sections and provided a METAVIR score following a protocol blinded to genotype.

### Immunofluorescence staining.

Liver tissue was fixed in 4% PFA and routinely processed for paraffin-embedding and cross-sectioned to obtain 5 μm–thick sections. Sections were dewaxed with toluene, then rehydrated with graded washes of ethanol, ending with water. Antigen retrieval was performed using sodium citrate buffer (0.1 M, pH 6, with 0.05% Tween-20) in a microwave, then washed with de-ionized water and PBS on a shaker. Sections were blocked with 10% donkey serum (catalog D9663 MilliporeSigma) for 30 minutes at room temperature. Antibodies against MARCO (Abcam, clone EPR22944-66, catalog ab259264, 1:200), F4/80 (Invitrogen, Thermo Fisher Scientific, clone CI:A3-1, catalog MA1-91124, 1:250), and CLEC4F (R&D Systems, Bio-Techne, catalog AF2784, 1:200) were incubated overnight at 4°C. Slides were washed with PBS-Tween, then incubated with secondary antibodies donkey anti-rabbit IgG (H+L) highly cross-absorbed Alexa Fluor Plus 647 (A32795, 1:500), donkey anti-rat IgG (H+L) highly cross-absorbed Alexa Fluor Plus 647 (A48272, 1:500), and donkey anti-goat IgG (H+L) cross-absorbed Alexa Fluor 555 (A-21432, 1:500) for 45 minutes at room temperature. Slides were washed and nuclei were stained using DAPI (Thermo Fisher Scientific 62248, 1:2,000) for 5 minutes at room temperature, washed, and mounted (Abcam, ab104135). Images were obtained on a Zeiss AxioImager Z1 epifluorescence microscope with 20× or 63× oil immersion objective and analyzed using Zeiss ZEN Blue microscopy software.

### Liver TG and cholesterol content.

Total liver lipids were extracted using a modified Folch method (92). For SLD- and HFHC-fed mice, a 100 mg or 50 mg piece of liver tissue was homogenized in 4 mL chloroform/methanol (2:1, v/v) and processed as described previously (93). Lipids were quantified using Infinity Cholesterol or Triglyceride reagent (both Thermo Fisher Scientific) at 540 nm.

### Hepatic F4/80^+^ cell isolation.

Fresh mouse livers were enzymatically digested using the components of a liver dissociation kit (kit 130-105-807, Miltenyi Biotec), and the gentleMACS Dissociators were used for the mechanical dissociation steps as previously described (94). Hepatic F4/80^+^ cells were isolated from dissociated liver samples with Anti-F4/80 MicroBeads UltraPure (mouse, Miltenyi Biotec) as per manufacturer’s protocol and flash-frozen in liquid nitrogen before being stored at –80°C for mRNA extraction.

### RNA isolation, cDNA, and qRT-PCR.

Frozen liver and isolated hepatic F4/80^+^ cells were homogenized with Tri Reagent (MilliporeSigma) using a TissueLyser II system (QIAGEN), and total RNA was extracted using manufacturer’s protocol. Reverse transcription was performed with the Applied Biosystems (Thermo Fisher Scientific) High-Capacity cDNA Reverse Transcription Kit. cDNA was subsequently used to assess mRNA expression by qRT-PCR (QuantStudio 5 System, Thermo Fisher Scientific) with TaqMan Fast Advanced Master Mix (Thermo Fisher Scientific, 4444557) and TaqMan Gene Expression Assays (Thermo Fisher Scientific). The specific gene expression assays used are listed (Supplemental Table 1). Quantification of transcript levels was performed by the standard curve method, and expression levels for each gene were normalized to *Actb* (β-actin).

### NanoString mRNA analysis.

NanoString Technologies nCounter Mouse Immunology Panel (catalog XT-CSO-MIM1-12) was used where 100 ng RNA was incubated with reporter and capture probes, consisting of 547 immunology-related mouse genes and 14 internal reference controls, for 16 hours at 65°C. Following hybridization, unbound probes were removed. According to the manufacturer’s instructions, assays were performed and quantified on the nCounter system, sample preparation station, and digital analyzer (NanoString Technologies).

Raw gene expression data were analyzed using NanoString’s software, nSolver v4.0.70, with the Advanced Analysis Module v2.0.115 with background subtraction. Genes with counts below a threshold of 20 were excluded from subsequent analysis. Data normalization was performed on background-subtracted samples using internal positive controls and selected housekeeping genes. Housekeeping genes were selected based on those that were consistent in all analyses across genotypes and diets: *Sdha*, *Eef1g*, *Gapdh*, *Hprt*, *Polr2a*, *Rpl19*, *Oaz1*, *Tbp*, and *Tubb5* for liver tissue and *Rpl19*, *Ppia*, *Oaz1*, *Eef1g*, *Sdha*, *Pol2a*, *Gusb*, *Tubb5*, *Gapdh*, and *Hprt* for F4/80^+^ cells. Differential gene expression analyses were performed using nSolver, which applies several multivariate linear regression models to identify significant genes (mixture negative binomial, simplified negative binomial, or log-linear model). Raw mRNA counts were log_2_-transformed, and significance was determined using 2-tailed *t* test. Statistically significant differentially expressed genes were identified as those with a *P* < 0.05. Ratios of log_2_-normalized transcript count data were generated for SLD *Dpp4^–/–^* mice versus baseline SLD WT mice, HFHC-fed *Dpp4^–/–^* mice versus baseline HFHC-fed WT mice, and HFHC-fed *Dpp4^hep–/–^* mice versus baseline HFHC-fed *Dpp4^GFP^* mice. The NanoString data have been deposited in NCBI’s Gene Expression Omnibus (GEO) and are accessible through GEO Series accession number GSE218767.

Pathway scores generated from nSolver Advanced Analysis were standardized by *Z*-scaling. ClustVis (95) was used to perform supervised hierarchical clustering analysis and principal component analysis of log_2_-transformed transcript count data and *Z*-scaled pathway scores.

### Human studies.

Participants were recruited between July 2015 and April 2016 from The Ottawa Hospital Viral Hepatitis Program (Ottawa, Canada). All participants were 18 years or older, planned to initiate HCV antiviral treatment, and provided signed informed consent documents to participate in a single-center, open-label study (ClinicalTrials.gov Identifier: NCT02734173), which was approved by The Ottawa Health Science Network Research Ethics Board (REB 2015-0305). Three groups of patients were examined for this study: noncirrhotic genotype 1a–infected participants receiving standard therapy plus ribavirin, noncirrhotic genotype 1b–infected participants receiving standard therapy, and compensated cirrhotic genotype 1a– or 1b–infected participants dosed with standard therapy plus ribavirin; all had a HOMA-IR ≥ 2 (58). Patients were treated for 12 weeks with ribavirin and direct-acting antivirals, after which they achieved sustained virologic response, and they were followed up for an additional 36 weeks. Inclusion and exclusion criteria, as well as methods for treatment, have been described (58). Plasma measurements were conducted by Laboratory Services at The Ottawa Hospital, as standard clinical procedure. Additional blood samples were treated with 1% Triton X-100 and 0.3% tributyl phosphate and incubated at 37°C for 1 hour to destroy any virus. The concentrations of circulating factors in treated plasma were quantified using multiplexing immunobead assays analyzed using Meso Scale Diagnostics as described above. Plasma DPP4 concentration was quantified using the R-PLEX Human DPPIV Antibody Set (Meso Scale Diagnostics, catalog F21YC) (96). To remove the variance in trait values attributed to sex and age differences for results described in Figure 7, a linear regression model compared the retrieved residuals (adjusted trait values) using the unpaired *t* test function available in R (version 4.0.5). Data are expressed as mean ± SD, and *P* < 0.05 was considered statistically significant.

### Statistics.

All data were plotted and statistical analyses were performed using GraphPad Prism (version 8.4.3). Data are expressed as mean ± SEM; human data are expressed as mean ± SD. Statistical differences between groups were evaluated by 2-way ANOVA with Tukey’s multiple-comparison post hoc test when analyzing time course data. All other differences were evaluated by a 2-tailed unpaired *t* test with Welch’s correction. *P* < 0.05 was considered statistically significant.

### Study approval.

Animals were cared for in accordance with the Canadian *Guide to the Care and Use of Laboratory Animals* (Canadian Council on Animal Care, 2020). Experimental procedures were approved under AUP#2909 and AUP#2029 by the University of Ottawa Animal Care and Veterinary Service (Ottawa, Ontario, Canada). Experiments in *Pemt^+/+^* and *Pemt*^−*/*−^ were approved by the University of Alberta’s Institutional Animal Care Committee (Edmonton, Alberta, Canada).

All participants were 18 years or older, planned to initiate HCV antiviral treatment, and provided written informed consent to participate in a single-center, open-label study (ClinicalTrials.gov Identifier: NCT02734173), which was approved by The Ottawa Health Science Network Research Ethics Board (REB 2015-0305, Ottawa, Ontario, Canada).

## Author contributions

MAD, MDF, and EEM conceptualized the study; KHK, MDF, and EEM developed methodology; NAT performed formal analysis, NAT, BV, MAN, NJ, EF, NMM, CAAL, NT, AAH, JRCN, CO, JNVDV, ILS, RS, SMP, ACC, AMC, RLJ, MAD, CLC, KHK, MDF, and EEM investigated; MDF and EEM provided resources; NAT, BV, and EEM wrote the original draft; all authors reviewed and edited the draft; KHK, MDF, EEM, and RLJ supervised the project; and EEM performed project administration and funding acquisition.

## Supplementary Material

Supplemental data

## Version 1 12/06/2022

In-Press Preview

## Version 2 01/24/2023

Electronic publication

## References

[1] Haedersdal S (2018). The role of glucagon in the pathophysiology and treatment of type 2 diabetes. Mayo Clin Proc.

[2] Petersen MC, Shulman GI (2018). Mechanisms of insulin action and insulin resistance. Physiol Rev.

[3] Eslam M (2020). MAFLD: a consensus-driven proposed nomenclature for metabolic associated fatty liver disease. Gastroenterology.

[4] McLean BA (2021). Revisiting the complexity of GLP-1 action from sites of synthesis to receptor activation. Endocr Rev.

[5] El K, Campbell JE (2020). The role of GIP in α-cells and glucagon secretion. Peptides.

[6] El K (2021). GIP mediates the incretin effect and glucose tolerance by dual actions on α cells and β cells. Sci Adv.

[7] Nauck M (1986). Reduced incretin effect in type 2 (non-insulin-dependent) diabetes. Diabetologia.

[8] Junker AE (2016). Diabetic and nondiabetic patients with nonalcoholic fatty liver disease have an impaired incretin effect and fasting hyperglucagonaemia. J Intern Med.

[9] Sunny NE (2011). Excessive hepatic mitochondrial TCA cycle and gluconeogenesis in humans with nonalcoholic fatty liver disease. Cell Metab.

[10] Deacon CF (2020). Dipeptidyl peptidase 4 inhibitors in the treatment of type 2 diabetes mellitus. Nat Rev Endocrinol.

[11] Mulvihill EE (2018). Dipeptidyl peptidase inhibitor therapy in type 2 diabetes: control of the incretin axis and regulation of postprandial glucose and lipid metabolism. Peptides.

[12] Mulvihill EE, Drucker DJ (2014). Pharmacology, physiology and mechanisms of action of dipeptidyl peptidase-4 inhibitors. Endocr Rev.

[13] Lamers D (2011). Dipeptidyl peptidase 4 is a novel adipokine potentially linking obesity to the metabolic syndrome. Diabetes.

[14] Sarkar J (2019). Increased plasma dipeptidyl peptidase-4 (DPP4) activity is an obesity-independent parameter for glycemic deregulation in type 2 diabetes patients. Front Endocrinol (lausanne).

[15] Grungreiff K (2009). Plasma concentrations of zinc, copper, interleukin-6 and interferon-γ, and plasma dipeptidyl peptidase IV activity in chronic hepatitis C. Mol Med Rep.

[16] Itou M (2008). Altered expression of glucagon-like peptide-1 and dipeptidyl peptidase IV in patients with HCV-related glucose intolerance. J Gastroenterol Hepatol.

[17] Varga T (2011). Higher serum DPP-4 enzyme activity and decreased lymphocyte CD26 expression in type 1 diabetes. Pathol Oncol Res.

[18] Mannucci E (2005). Hyperglycaemia increases dipeptidyl peptidase IV activity in diabetes mellitus. Diabetologia.

[19] Ryskjaer J (2006). Plasma dipeptidyl peptidase-IV activity in patients with type-2 diabetes mellitus correlates positively with HbAlc levels, but is not acutely affected by food intake. Eur J Endocrinol.

[20] Miyazaki M (2012). Increased hepatic expression of dipeptidyl peptidase-4 in non-alcoholic fatty liver disease and its association with insulin resistance and glucose metabolism. Mol Med Rep.

[21] Baumeier C (2017). Hepatic DPP4 DNA methylation associates with fatty liver. Diabetes.

[22] Varin EM (2019). Circulating levels of soluble dipeptidyl peptidase-4 are dissociated from inflammation and induced by enzymatic DPP4 inhibition. Cell Metab.

[23] Gorgens SW (2019). A siRNA mediated hepatic dpp4 knockdown affects lipid, but not glucose metabolism in diabetic mice. PLoS One.

[24] Ghorpade DS (2018). Hepatocyte-secreted DPP4 in obesity promotes adipose inflammation and insulin resistance. Nature.

[25] Baumeier C (2017). Elevated hepatic DPP4 activity promotes insulin resistance and non-alcoholic fatty liver disease. Mol Metab.

[26] Balas B (2007). The dipeptidyl peptidase IV inhibitor vildagliptin suppresses endogenous glucose production and enhances islet function after single-dose administration in type 2 diabetic patients. J Clin Endocrinol Metab.

[27] Carris NW, Dietrich EA (2022). Semaglutide for weight loss. Ann Pharmacother.

[28] Da BL, Satapathy SK (2021). Semaglutide or placebo for nonalcoholic steatohepatitis. N Engl J Med.

[29] Meier JJ (2021). Efficacy of semaglutide in a subcutaneous and an oral formulation. Front Endocrinol (lausanne).

[30] Slawson DC (2021). Once-weekly semaglutide is an effective adjunct for weight loss in adults without diabetes who are overweight or obese. Am Fam Physician.

[31] Srivastava G, Kumar RB (2021). Once-weekly semaglutide in adults with overweight or obesity. N Engl J Med.

[32] Frias JP (2017). The sustained effects of a dual GIP/GLP-1 receptor agonist, NNC0090-2746, in patients with type 2 diabetes. Cell Metab.

[33] Jall S (2017). Monomeric GLP-1/GIP/glucagon triagonism corrects obesity, hepatosteatosis, and dyslipidemia in female mice. Mol Metab.

[34] Muscelli E (2012). Mechanisms for the antihyperglycemic effect of sitagliptin in patients with type 2 diabetes. J Clin Endocrinol Metab.

[35] Serre V (1998). Exendin-(9-39) is an inverse agonist of the murine glucagon-like peptide-1 receptor: implications for basal intracellular cyclic adenosine 3′,5′-monophosphate levels and β-cell glucose competence. Endocrinology.

[36] D’Alessio D (2007). Fasting and postprandial concentrations of GLP-1 in intestinal lymph and portal plasma: evidence for selective release of GLP-1 in the lymph system. Am J Physiol Regul Integr Comp Physiol.

[37] Qin X (2005). GLP-1 reduces intestinal lymph flow, triglyceride absorption, and apolipoprotein production in rats. Am J Physiol Gastrointest Liver Physiol.

[38] Mulvihill EE (2017). Cellular sites and mechanisms linking reduction of dipeptidyl peptidase-4 activity to control of incretin hormone action and glucose homeostasis. Cell Metab.

[39] Buzzetti E (2016). The multiple-hit pathogenesis of non-alcoholic fatty liver disease (NAFLD). Metabolism.

[40] Williams KH (2015). Circulating dipeptidyl peptidase-4 activity correlates with measures of hepatocyte apoptosis and fibrosis in non-alcoholic fatty liver disease in type 2 diabetes mellitus and obesity: a dual cohort cross-sectional study. J Diabetes.

[41] Wree A (2014). NLRP3 inflammasome activation results in hepatocyte pyroptosis, liver inflammation, and fibrosis in mice. Hepatology.

[42] Wada N (2017). Serum-inducible protein (IP)-10 is a disease progression-related marker for non-alcoholic fatty liver disease. Hepatol Int.

[43] Berres ML (2010). Antagonism of the chemokine Ccl5 ameliorates experimental liver fibrosis in mice. J Clin Invest.

[44] Papatheodoridi AM (2020). The role of senescence in the development of nonalcoholic fatty liver disease and progression to nonalcoholic steatohepatitis. Hepatology.

[45] Kim KM (2017). Identification of senescent cell surface targetable protein DPP4. Genes Dev.

[46] Lopes-Paciencia S (2019). The senescence-associated secretory phenotype and its regulation. Cytokine.

[47] Medeiros Tavares Marques JC (2017). Identification of new genes associated to senescent and tumorigenic phenotypes in mesenchymal stem cells. Sci Rep.

[48] el-Deiry WS (1993). WAF1, a potential mediator of p53 tumor suppression. Cell.

[49] Kazankov K (2019). The role of macrophages in nonalcoholic fatty liver disease and nonalcoholic steatohepatitis. Nat Rev Gastroenterol Hepatol.

[50] Weiskirchen R (2019). Organ and tissue fibrosis: molecular signals, cellular mechanisms and translational implications. Mol Aspects Med.

[51] Zhong J (2013). A potential role for dendritic cell/macrophage-expressing DPP4 in obesity-induced visceral inflammation. Diabetes.

[52] Romacho T (2020). DPP4 deletion in adipose tissue improves hepatic insulin sensitivity in diet-induced obesity. Am J Physiol Endocrinol Metab.

[53] Lee YS (2019). Hepatocyte-specific HIF-1α ablation improves obesity-induced glucose intolerance by reducing first-pass GLP-1 degradation. Sci Adv.

[54] Jacobs RL (2010). Impaired de novo choline synthesis explains why phosphatidylethanolamine N-methyltransferase-deficient mice are protected from diet-induced obesity. J Biol Chem.

[55] Gao X (2015). Decreased lipogenesis in white adipose tissue contributes to the resistance to high fat diet-induced obesity in phosphatidylethanolamine N-methyltransferase-deficient mice. Biochim Biophys Acta.

[56] Itou M (2013). Dipeptidyl peptidase-4: a key player in chronic liver disease. World J Gastroenterol.

[57] Chang ML (2016). Metabolic alterations and hepatitis C: from bench to bedside. World J Gastroenterol.

[58] Doyle MA (2019). Hepatitis C direct acting antivirals and ribavirin modify lipid but not glucose parameters. Cells.

[59] Maes M (2001). Treatment with interferon-alpha (IFN alpha) of hepatitis C patients induces lower serum dipeptidyl peptidase IV activity, which is related to IFN alpha-induced depressive and anxiety symptoms and immune activation. Mol Psychiatry.

[60] Meissner EG (2015). Dynamic changes of post-translationally modified forms of CXCL10 and soluble DPP4 in HCV subjects receiving interferon-free therapy. PLoS One.

[61] Baggio LL, Drucker DJ (2021). Glucagon-like peptide-1 receptor co-agonists for treating metabolic disease. Mol Metab.

[62] Drucker DJ (2018). Mechanisms of action and therapeutic application of glucagon-like peptide-1. Cell Metab.

[63] Burcelin R (2001). Glucose competence of the hepatoportal vein sensor requires the presence of an activated glucagon-like peptide-1 receptor. Diabetes.

[64] Aulinger BA (2020). Rapid hepatic metabolism blunts the endocrine action of portally infused GLP-1 in male rats. Am J Physiol Endocrinol Metab.

[65] Trzaskalski NA (2020). Dipeptidyl peptidase-4 at the interface between inflammation and metabolism. Clin Med Insights Endocrinol Diabetes.

[66] Jejelava N (2018). Intestinal lymph as a readout of meal-induced GLP-1 release in an unrestrained rat model. Am J Physiol Regul Integr Comp Physiol.

[67] Shin JW (2008). Lymphatic-specific expression of dipeptidyl peptidase IV and its dual role in lymphatic endothelial function. Exp Cell Res.

[68] Malbert CH (2021). Glucose sensing mediated by portal glucagon-like peptide 1 receptor is markedly impaired in insulin-resistant obese animals. Diabetes.

[69] Barchetta I (2021). Circulating dipeptidyl peptidase-4 is independently associated with the presence and severity of NAFLD/NASH in individuals with and without obesity and metabolic disease. J Endocrinol Invest.

[70] Kawakubo M (2020). Dipeptidyl peptidase-4 inhibition prevents nonalcoholic steatohepatitis-associated liver fibrosis and tumor development in mice independently of its anti-diabetic effects. Sci Rep.

[71] Kaji K (2014). Dipeptidyl peptidase-4 inhibitor attenuates hepatic fibrosis via suppression of activated hepatic stellate cell in rats. J Gastroenterol.

[72] Koyama Y, Brenner DA (2017). Liver inflammation and fibrosis. J Clin Invest.

[73] Guo Y (2020). NF- κ B/HDAC1/SREBP1c pathway mediates the inflammation signal in progression of hepatic steatosis. Acta Pharm Sin B.

[74] Dela Pena A (2005). NF-kappaB activation, rather than TNF, mediates hepatic inflammation in a murine dietary model of steatohepatitis. Gastroenterology.

[75] Zeng Y (2014). The DPP-4 inhibitor sitagliptin attenuates the progress of atherosclerosis in apolipoprotein-E-knockout mice via AMPK- and MAPK-dependent mechanisms. Cardiovasc Diabetol.

[76] Shinjo T (2015). DPP-IV inhibitor anagliptin exerts anti-inflammatory effects on macrophages, adipocytes, and mouse livers by suppressing NF-κB activation. Am J Physiol Endocrinol Metab.

[77] Meijnikman AS (2021). Evaluating causality of cellular senescence in non-alcoholic fatty liver disease. JHEP Rep.

[78] Chen Z (2020). Dipeptidyl peptidase-4 inhibition improves endothelial senescence by activating AMPK/SIRT1/Nrf2 signaling pathway. Biochem Pharmacol.

[79] Diaz-Jimenez D (2020). Glucocorticoids mobilize macrophages by transcriptionally up-regulating the exopeptidase DPP4. J Biol Chem.

[80] Wang T (2021). Targeting cellular senescence prevents glucocorticoid-induced bone loss through modulation of the DPP4-GLP-1 axis. Signal Transduct Target Ther.

[81] Rufini A (2013). Senescence and aging: the critical roles of p53. Oncogene.

[82] Xie Y (2017). The tumor suppressor p53 limits ferroptosis by blocking DPP4 activity. Cell Rep.

[83] Svendsen B (2018). Insulin secretion depends on intra-islet glucagon signaling. Cell Rep.

[84] Capozzi ME (2019). β cell tone is defined by proglucagon peptides through cAMP signaling. JCI Insight.

[85] Zhu L (2019). Intra-islet glucagon signaling is critical for maintaining glucose homeostasis. JCI Insight.

[86] Song Y (2019). Gut-proglucagon-derived peptides are essential for regulating glucose homeostasis in mice. Cell Metab.

[87] Chepurny OG (2019). Nonconventional glucagon and GLP-1 receptor agonist and antagonist interplay at the GLP-1 receptor revealed in high-throughput FRET assays for cAMP. J Biol Chem.

[88] Marguet D (2000). Enhanced insulin secretion and improved glucose tolerance in mice lacking CD26. Proc Natl Acad Sci U S A.

[89] Walkey CJ (1997). Disruption of the murine gene encoding phosphatidylethanolamine *N*-methyltransferase. Proc Natl Acad Sci U S A.

[90] Fullerton MD (2013). Single phosphorylation sites in Acc1 and Acc2 regulate lipid homeostasis and the insulin-sensitizing effects of metformin. Nat Med.

[91] Chambers AP (2017). The role of pancreatic preproglucagon in glucose homeostasis in mice. Cell Metab.

[92] Folch J (1957). A simple method for the isolation and purification of total lipides from animal tissues. J Biol Chem.

[93] Patel R (2011). LXRβ is required for glucocorticoid-induced hyperglycemia and hepatosteatosis in mice. J Clin Invest.

[94] Rao X (2019). Oxidized LDL upregulates macrophage DPP4 expression via TLR4/TRIF/CD36 pathways. EBioMedicine.

[95] Metsalu T, Vilo J (2015). ClustVis: a web tool for visualizing clustering of multivariate data using principal component analysis and heatmap. Nucleic Acids Res.

[96] Baggio LL (2020). Plasma levels of DPP4 activity and sDPP4 are dissociated from inflammation in mice and humans. Nat Commun.

