# Supplementary material for: Hepatocyte-derived DPP4 regulates portal GLP-1 bioactivity, modulates glucose production, and when absent influences NAFLD progression
Source: JCI Insight. 2023 Jan 24;8(2):e154314. doi: 10.1172/jci.insight.154314 (PMC9977314; doi:10.1172/jci.insight.154314)
Supplement: Supplemental data [file jciinsight-8-154314-s112.pdf]

Supplementary Material

Hepatocyte-derived DPP4 regulates portal GLP-1 bioactivity, glucose production and its absence alters liver disease progression

Authors: Natasha A. Trzaskalski[1,2]*, Branka Vulesevic[1,2]*, My-Anh Nguyen[1,2], Natasha Jeraj[1,2], Evgenia Fadzeyeva[1,2], Nadya M. Morrow[1,2], Cassandra A. A. Locatelli[1,2], Nicole Travis[1,2], Antonio A. Hanson[1,2], Julia R.C. Nunes[1,3,4], Conor O'Dwyer[1,3,4], Jelske N. van der Veen[5], Ilka Lorenzen-Schmidt[2], Rick Seymour[2], Serena M. Pulente[1,2], Andrew C. Clément[1,2], Angela M. Crawley[1,3,6,7], René L. Jacobs[5], Mary Anne Doyle[8], Curtis L. Cooper[6,9], Kyoung-Han Kim[2,10], Morgan D. Fullerton[1,3,4], Erin E. Mulvihill[1,2,3,11]&

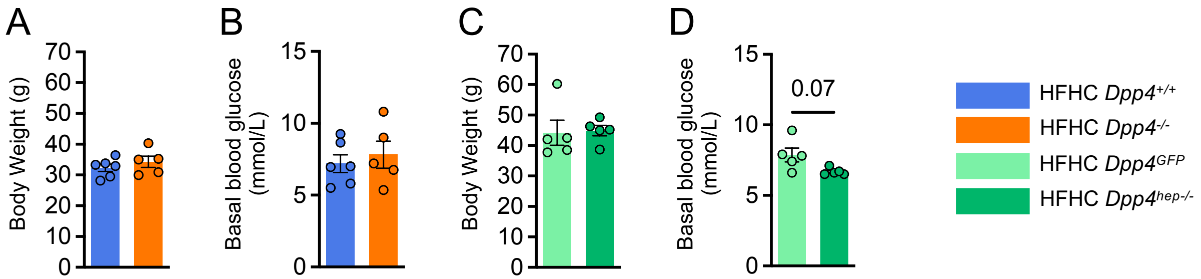

Supplementary Figure 1: Body weight and basal blood glucose in (A-B) HFHC-fed *Dpp4*$^{+/+}$ and *Dpp4*$^{-/-}$, and (C-D) *Dpp4*^{GFP} and *Dpp4*$^{hep-/-}$ mice, respectively, used for hyperinsulinemic-euglycemic clamp experiments. Data are presented as the means ± SEM, analyzed by unpaired students t-test with Welch's correction, ns p>0.05.

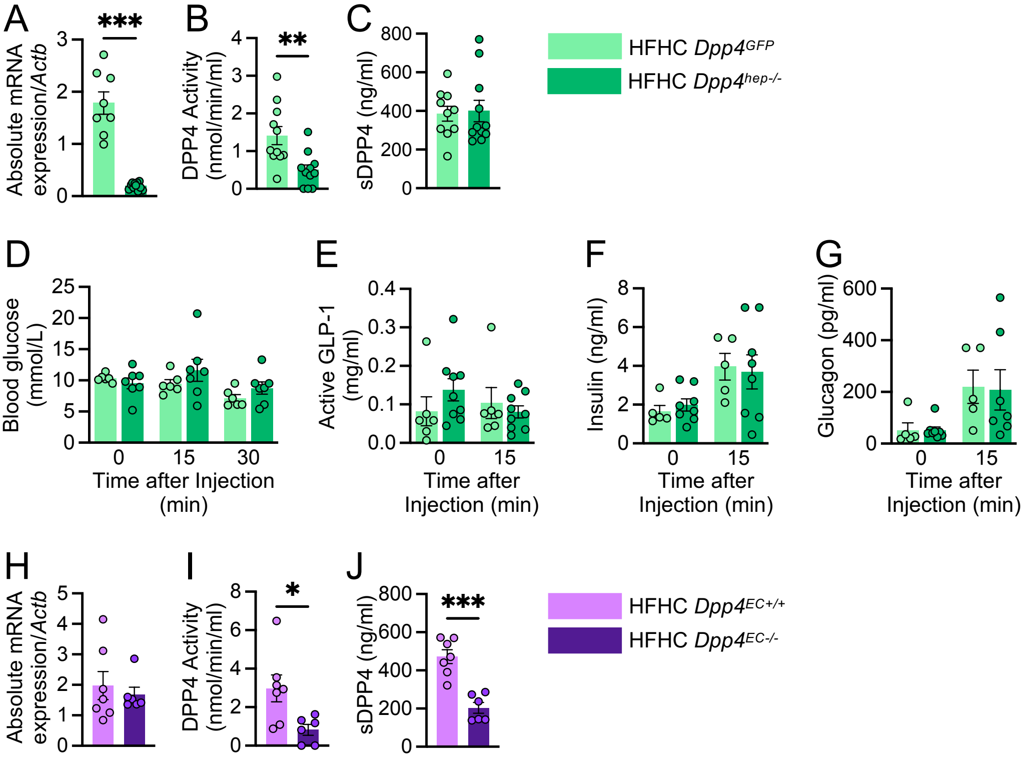

Supplementary Figure 2: (A) *Dpp4* mRNA abundance in the liver (relative to *Actb*), (B) fasting plasma DPP4 activity, and (C) plasma DPP4 protein in *Dpp4*^{GFP} and *Dpp4*$^{hep-/-}$ mice. (D) Blood glucose, (E) active GLP-1, (F) insulin and (G) glucagon during an arginine tolerance test at baseline, 15- and 30-minutes after intraperitoneal injection in *Dpp4*^{GFP} and *Dpp4*$^{hep-/-}$ mice fed a HFHC diet for 4 weeks. (H) *Dpp4* mRNA abundance in the liver (relative to *Actb*), (I) fasting plasma DPP4 activity and (J) plasma DPP4 protein in *Dpp4*$^{EC+/+}$ mice and *Dpp4*$^{EC-/-}$ mice. Data are presented as the means ± SEM, analyzed by unpaired students t-test with Welch's correction, ns p<0.05, *p=0.01-0.05, **p=0.001-0.01, ***p=0.0001-0.001 .

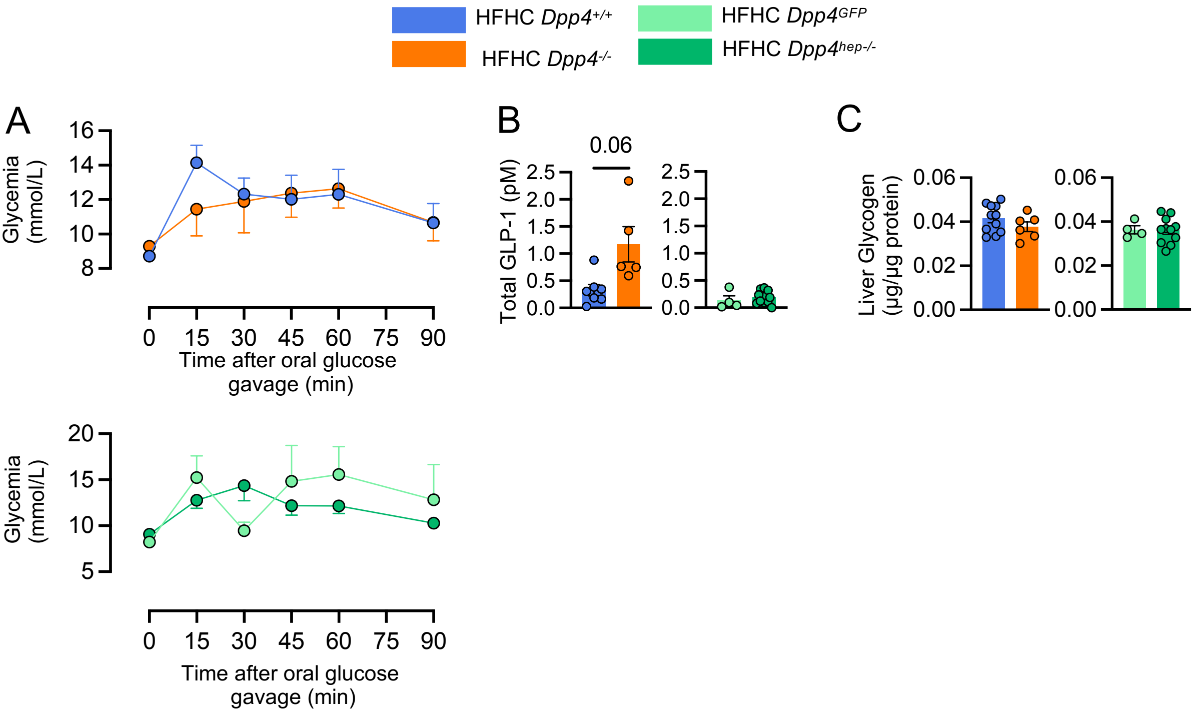

Supplementary Figure 3: (A) Oral glucose tolerance test in HFHC-fed *Dpp4*$^{+/+}$ and *Dpp4*$^{-/-}$ mice and HFHC-fed *Dpp4*^{GFP} and *Dpp4*$^{hep-/-}$ mice. (B) Total GLP-1 15-minutes post-oral glucose gavage in HFHC-fed *Dpp4*$^{+/+}$ and *Dpp4*$^{-/-}$ mice and HFHC-fed *Dpp4*^{GFP} and *Dpp4*$^{hep-/-}$ mice. (C) Liver glycogen content in HFHC-fed mice after 24 weeks. Data are presented as the means ± SEM. Time-course data is analyzed by two-way ANOVA with Tukey's multiple comparisons post hoc test, remaining data analyzed by unpaired students t-test with Welch's correction, ns $p<0.05$.

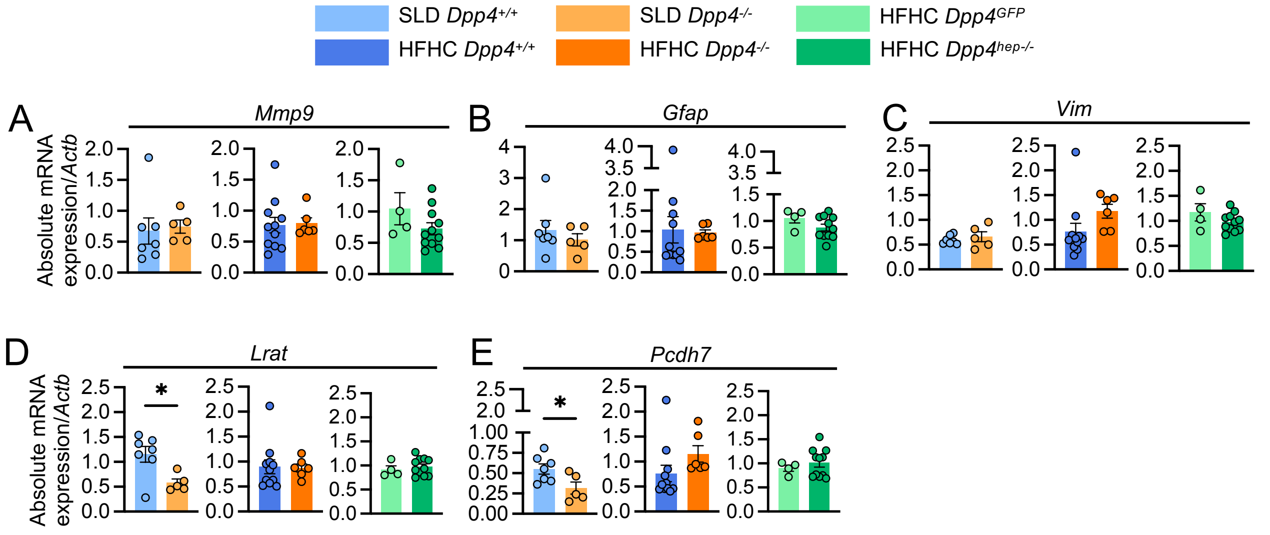

Supplementary Figure 4: Liver mRNA abundance (relative to *Actb*) for (A) *Mmp9*, (B) *Gfap*, (C) *Vim,* (D) *Lrat* and (E) *Pcdh7*. Data are presented as the means ± SEM, analyzed by unpaired students t-test with Welch's correction, ns $p<0.05$, *$p=0.01-0.05$.

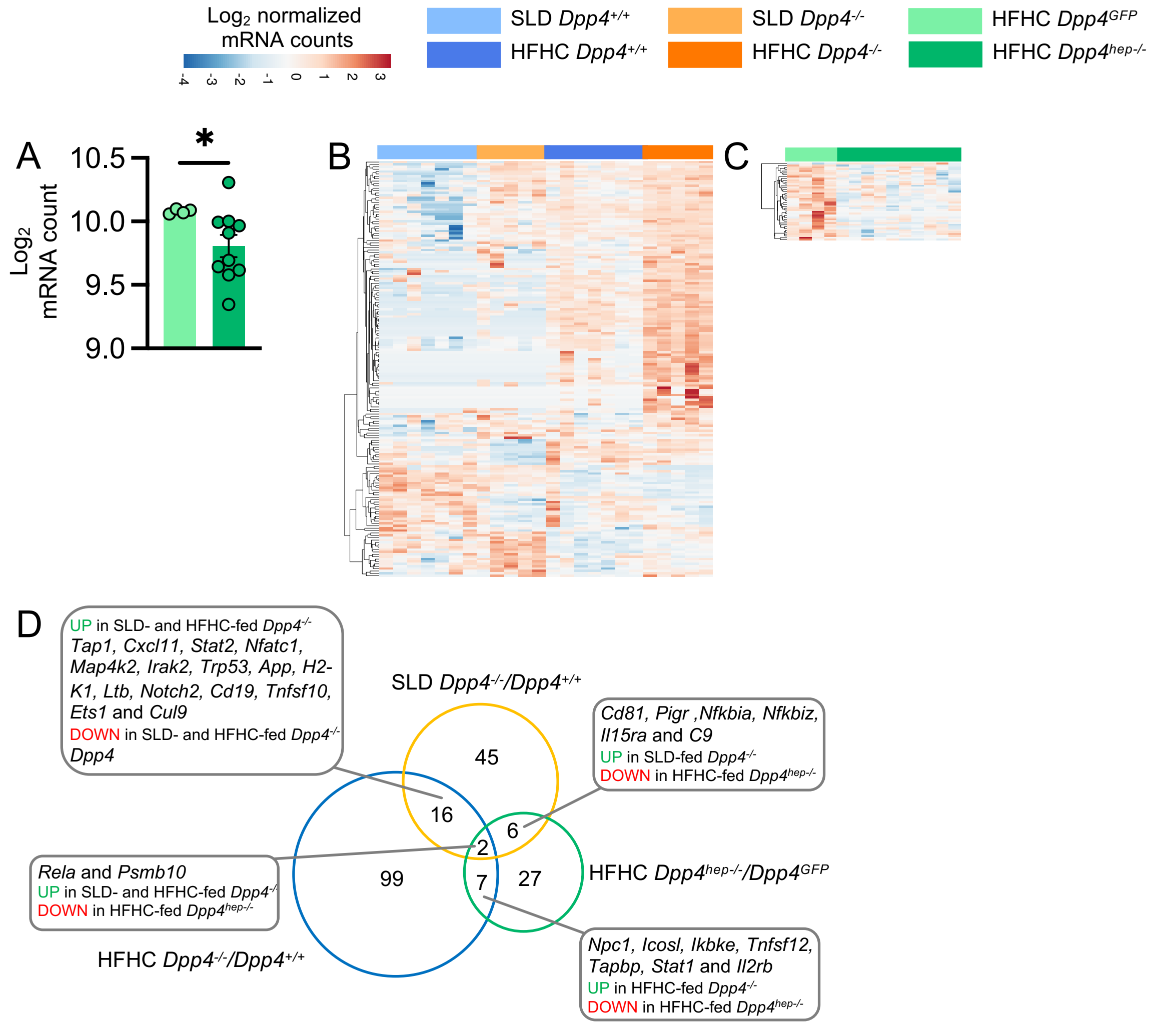

Supplementary Figure 5: (A) Log2 mRNA count of DPP4 in liver tissue. Supervised hierarchical cluster analysis of significantly differentially expressed genes in liver tissue from (B) SLD- and HFHC-fed *Dpp4*$^{+/+}$ and *Dpp4*$^{-/-}$ mice, and (C) *Dpp4*^{GFP} and *Dpp4*$^{hep-/-}$ mice, using the correlation distance method with average linkage, coloured by the $\log_2$ normalized mRNA count. (F) Venn diagram depicting number of gene identified as significantly differentially expressed in each comparison and overlap. Data are presented as the means ± SEM, analyzed by unpaired students t-test with Welch’s correction, ns p>0.05 and *p=0.01-0.05.

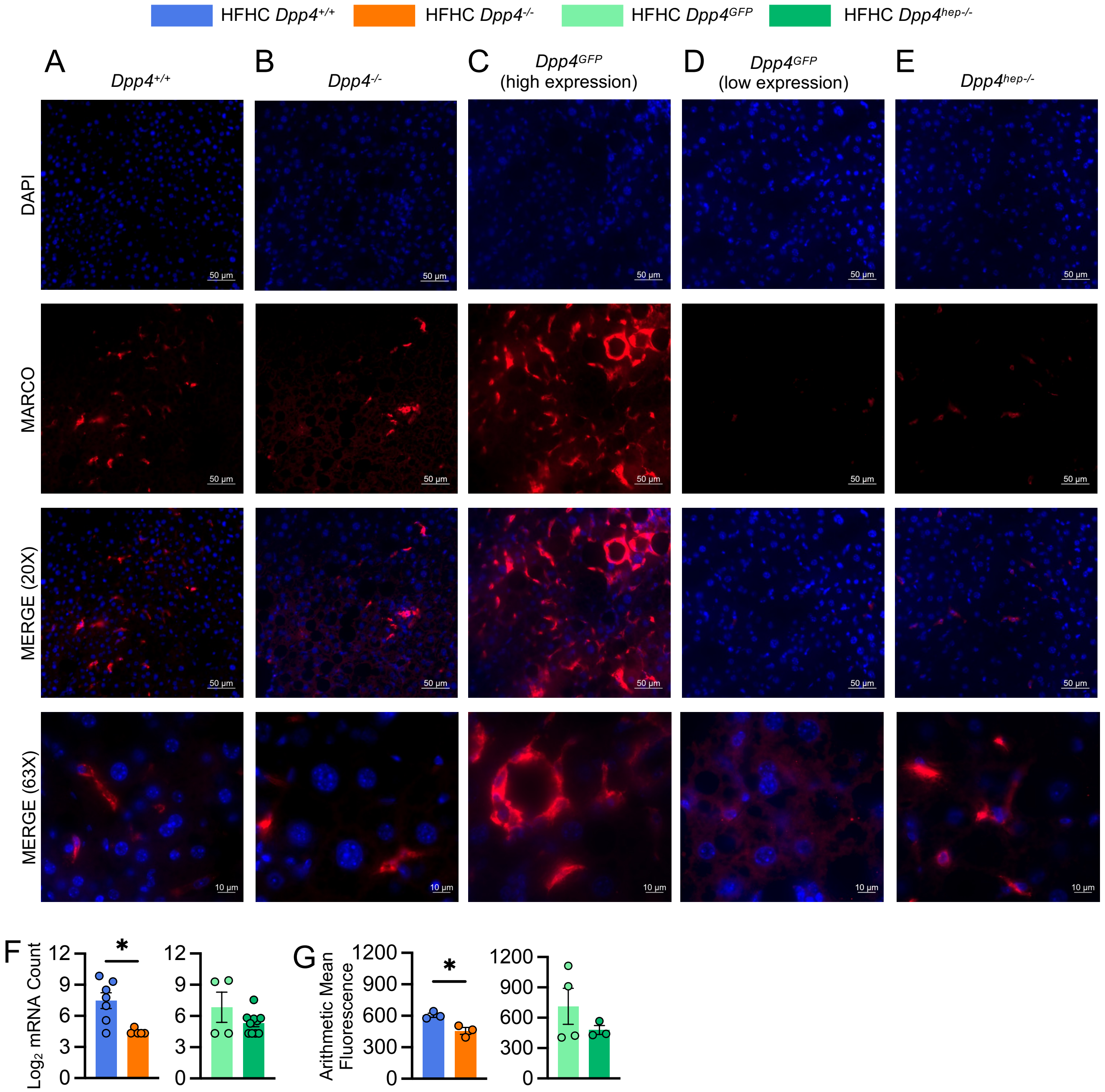

Supplementary Figure 6: Representative immunofluorescent images of MARCO in liver at 20X and 63X magnification for (A) HFHC-fed *Dpp4*$^{+/+}$, (B) HFHC-fed *Dpp4*$^{-/-}$, (C) HFHC-fed *Dpp4*^{GFP} (high expression sample), (D) HFHC-fed *Dpp4*^{GFP} (low expression sample) and (E) *Dpp4*$^{hep-/-}$ mice (scale bar at 20X = 50um, scale bar at 63X = 10um; 6 regions of interest, n=3-4). (F) NanoString log2 mRNA count for Marco (n=4-11). (G) Arithmetic mean fluorescence of MARCO. Data are presented as the means ± SEM, analyzed by unpaired students t-test with Welch's correction, ns p>0.05, *p=0.01-0.05.

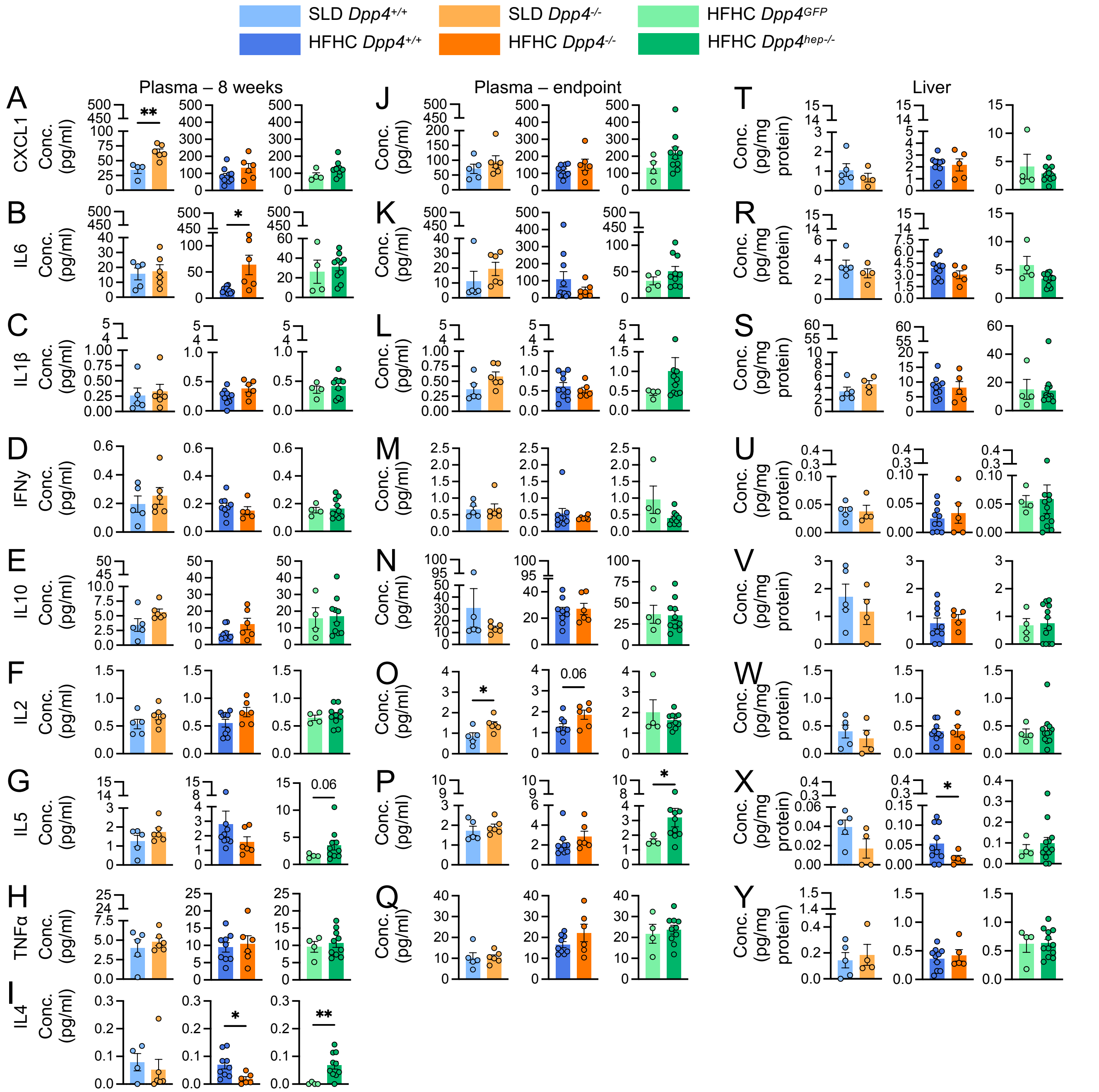

Supplementary Figure 7: Plasma concentration of chemokines in cytokines at (A-I) 8 weeks in plasma, (J-Q) endpoint in plasma and (R-Y) in liver tissue homogenates. Data are presented as the mean ± SEM, analyzed by unpaired students t-test with Welch's correction, *p=0.01-0.05, **p=0.001-0.01.

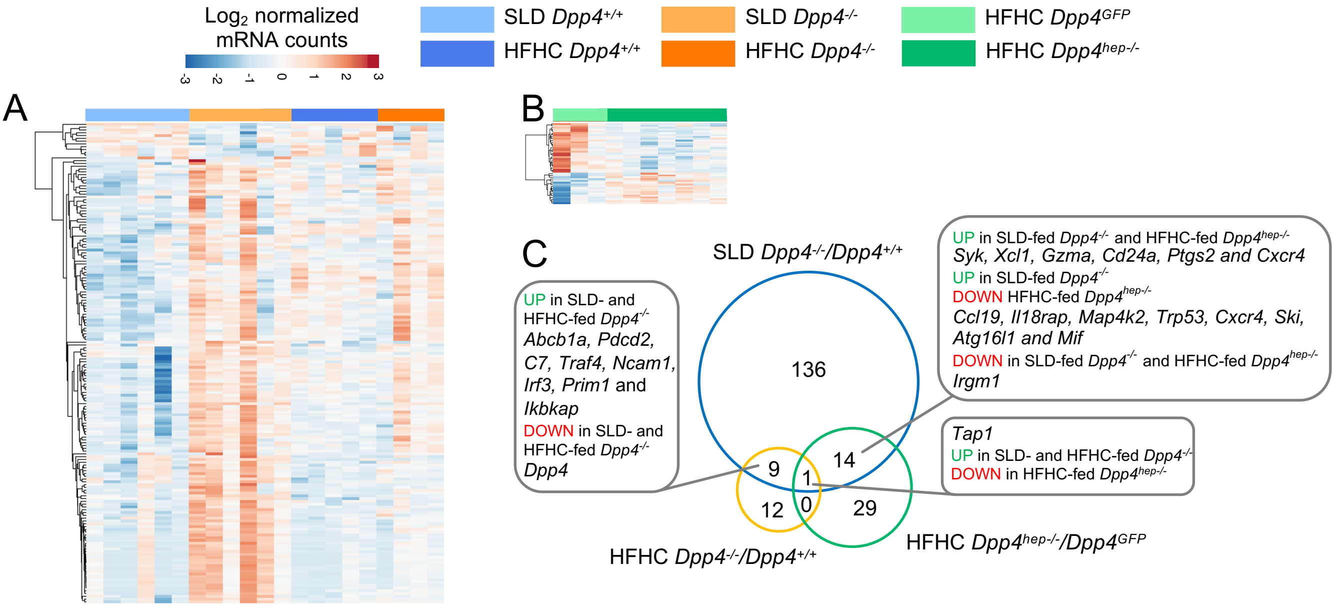

Supplementary Figure 8: Supervised hierarchical cluster analysis of significantly differentially expressed genes in F4/80+ cells isolated from liver tissue from (A) SLD- and HFHC-fed *Dpp4*$^{+/+}$ and *Dpp4*$^{-/-}$ mice, and (B) *Dpp4*^{GFP} and *Dpp4*$^{hep-/-}$ mice, using the correlation distance method with average linkage, coloured by the log_2 normalized mRNA count. (C) Venn diagram depicting number of genes identified as significantly differentially expressed in each comparison and overlap.

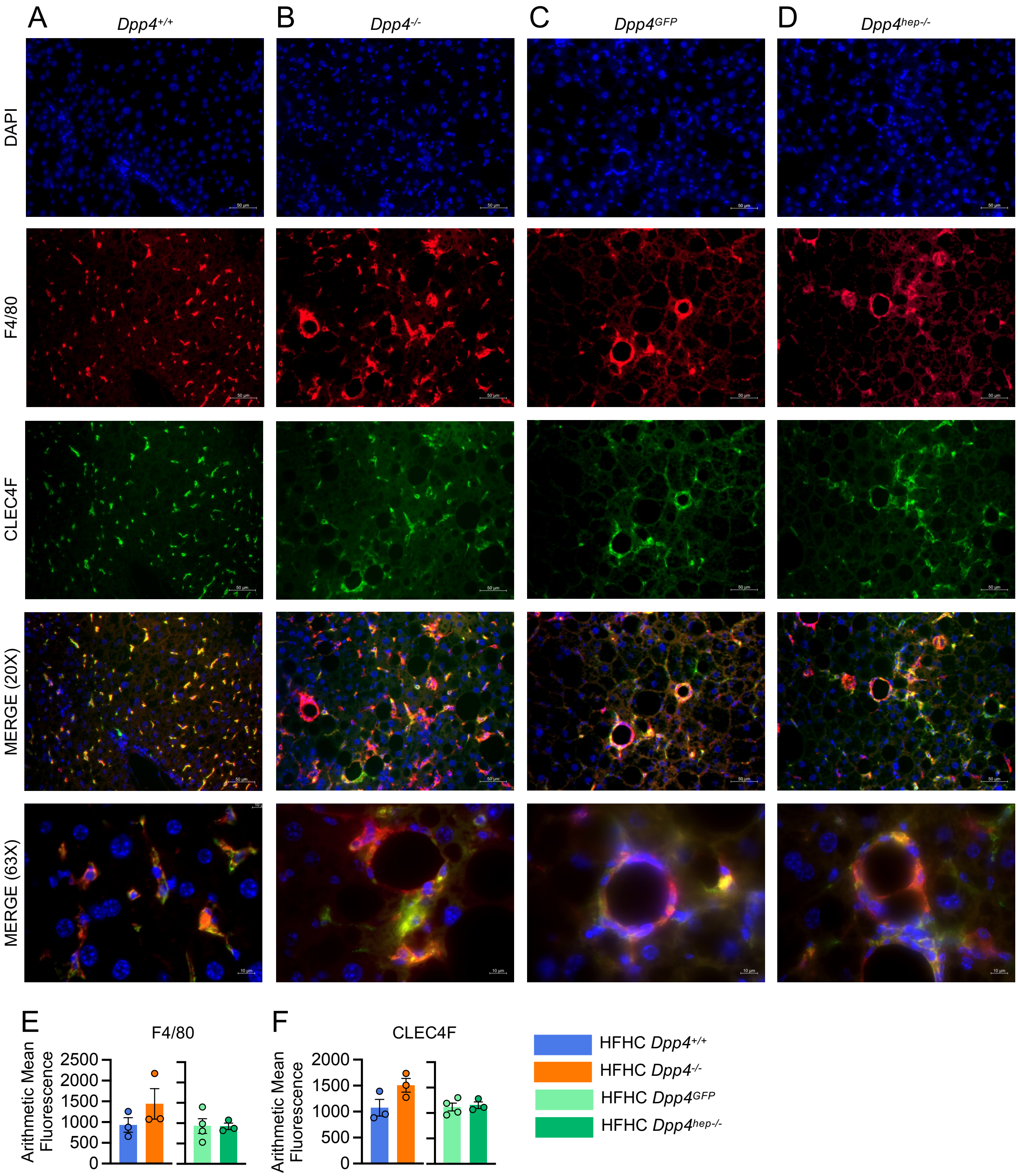

Supplementary Figure 9: Representative immunofluorescent images of F4/80 and CLEC4F in liver at 20X and 63X magnification for (A) HFHC-fed *Dpp4*$^{+/+}$, (B) HFHC-fed *Dpp4*$^{-/-}$, (C) HFHC-fed *Dpp4*^{GFP} and (D) *Dpp4*$^{hep-/-}$ mice (scale bar at 20X = 50um, scale bar at 63X = 10um; 6 regions of interest, n=3-4). Arithmetic mean fluorescence of (E) F4/80 and (F) CLEC4F. Data are presented as the means ± SEM, analyzed by unpaired students t-test with Welch's correction, ns p>0.05.

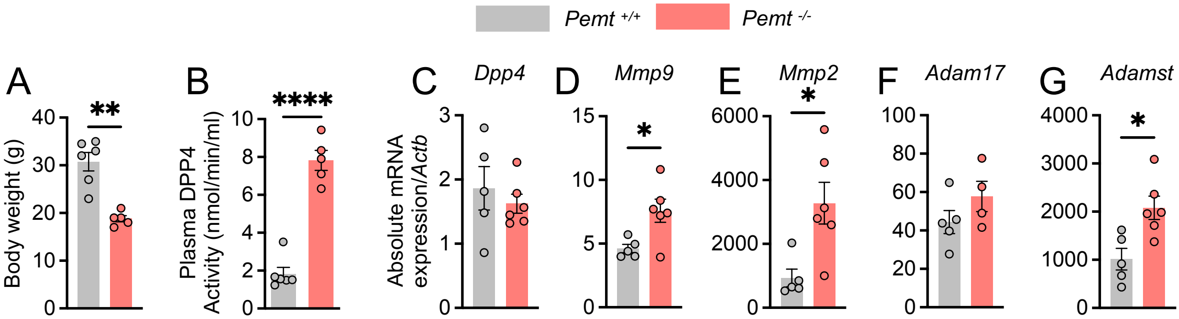

Supplementary Figure 10: (A) Body weight, (B) plasma DPP4 activity. Liver mRNA abundance of (C) *Dpp4*, (D) *Mmp9*, (E) *Mmp2*, (F) *Adam17* and (G) *Adamst* in *Pemt* $^{+/+}$ or *Pemt* $^{-/-}$ mice. Data are presented as the means ± SEM, analyzed by unpaired students t-test with Welch's correction, ns p>0.05, *p=0.01-0.05, **p=0.001-0.01, ***p=0.0001-0.001 and ****p<0.0001.

	Dpp4^GFP	*Dpp4^hep-/-*
Fasting plasma DPP4 activity (nmol/min/ml)	0.86	0.38
Fasting plasma DPP4 concentration (ng/ml)	250.50	370.50
Absolute *Dpp4* mRNA Expression/*Actb*	1.24	0.17
PV plasma DPP4 concentration (ng/ml)	159.50	185.50
PV plasma active GLP-1 (mg/ml)	1.29	4.55
CP plasma active GLP-1 (mg/ml)	0.18	0.53
PV plasma active GIP (pM)	6.33	27.17
CP plasma active GIP (pM)	1.50	5.17
PV plasma insulin (ng/ml)	5.37	3.47
CP plasma insulin (ng/ml)	2.31	2.77
PV plasma glucagon (pg/ml)	86.54	32.63
CP plasma glucagon (pg/ml)	10.20	12.15

Supplementary Table 1: Fasting and fed, plasma and liver DPP4 activity, concentration, active GLP-1, active GIP, insulin and glucagon from the portal vein (PV) or cardiac puncture (CP) 15 minutes after glucose bolus in aged (60 weeks), HFHC-fed *Dpp4^{GFP}* and *Dpp4$^{hep-/-}$* mice. Values are the mean (n=2/genotype)
